# Layered Double Hydroxide Nanosheets Incorporated Hierarchical Hydrogen Bonding Polymer Networks for Transparent and Fire-Proof Ceramizable Coatings

**DOI:** 10.1007/s40820-025-01646-y

**Published:** 2025-01-27

**Authors:** Bifan Guo, Yimin He, Yongming Chen, Tianci Yang, Chaohua Peng, Weiang Luo, Birong Zeng, Yiting Xu, Lizong Dai

**Affiliations:** 1 Fujian Provincial Key Laboratory of Fire Retardant Materials, College of Materials, Xiamen University, Xiamen, 361000 People’s Republic of China; 2 Xiamen Key Laboratory of Fire Retardant Materials, Xiamen University, Xiamen, 361000 People’s Republic of China

**Keywords:** Nanocomposites, Supramolecular, Flame retardancy, Ceramic-like char layer, Fire protection

## Abstract

In recent decades, annual urban fire incidents, including those involving ancient wooden buildings burned, transportation, and solar panels, have increased, leading to significant loss of human life and property. Addressing this issue without altering the surface morphology or interfering with optical behavior of flammable materials poses a substantial challenge. Herein, we present a transparent, low thickness, ceramifiable nanosystem coating composed of a highly adhesive base (poly(SSS_1_-co-HEMA_1_)), nanoscale layered double hydroxide sheets as ceramic precursors, and supramolecular melamine di-borate as an accelerator. We demonstrate that this hybrid coating can transform into a porous, fire-resistant protective layer with a highly thermostable vitreous phase upon exposure to flame/heat source. A nanosystem coating of just ~ 100 μm thickness can significantly increase the limiting oxygen index of wood (Pine) to 37.3%, dramatically reduce total heat release by 78.6%, and maintain low smoke toxicity (CIT_G_ = 0.016). Detailed molecular force analysis, combined with a comprehensive examination of the underlying flame-retardant mechanisms, underscores the effectiveness of this coating. This work offers a strategy for creating efficient, environmentally friendly coatings with fire safety applications across various industries.

**Supplementary Information:**

The online version contains supplementary material available at 10.1007/s40820-025-01646-y.

## Highlights

- A transparent and ceramizable coating was developed by incorporating nano-layered double hydroxide nanosheets into hierarchical hydrogen bonding polymer networks.
- The resulting coating composites demonstrated excellent high-temperature stability and fire resistance, effectively withstanding the direct exposure to a butane flame (~ 1100 °C) in air atmosphere.
- The mechanisms behind the flame-retardant behavior and ceramicization behaviors were thoroughly investigated and explained.

**Supplementary Information:**

The online version contains supplementary material available at 10.1007/s40820-025-01646-y.

## Graphical abstract

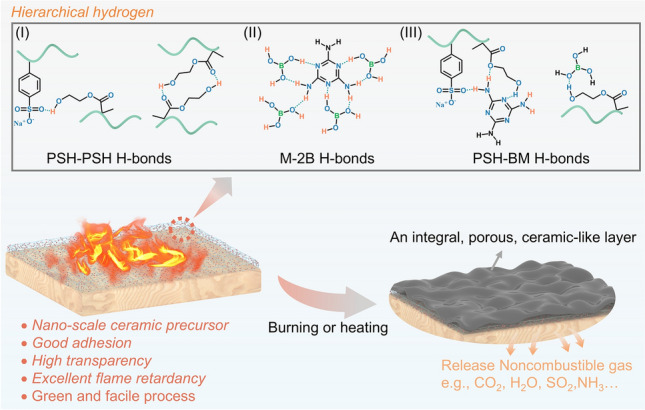

## Introduction

Fire, a cornerstone of civilization, has dramatically shaped the trajectory of human development, facilitating the transition of our ancestors from primitive to advanced societies [1]. Nevertheless, flames can quickly lead to devastating fire disasters, causing irretrievable losses of property and life once they are out of bounds [2]. Indeed, both human society and the natural world experience numerous fire incidents each year, with solid wood and various polymer products often serving as fuel sources [3, 4]. For example, the Notre-Dame de Paris fire in 2019 highlighted the vulnerability of wooden structures to fire, resulting in severe damages to this historic architecture [5]. On the other hand, the buildings incorporating photovoltaic are receiving increased attention due to the prevalence of green lifestyle [6]. However, these systems pose potential fire risks due to electrical arcing [7], which can release toxic chemicals and explosive gases [8]. As a result of such events, it is imperative to develop reliable and effective fire-retardant coating for flammable wooden products and other materials to ensure safety and protect lives.

In general, the design of fire-proof materials follows two main strategies: mechanical mixing with fire retardants and surface fire-retardant coating techniques. While the incorporation of traditional fire-retardant fillers (e.g., layered double hydroxide (LDHs) [9], and ammonium polyphosphate (APP) [10]) into a matrix through physical mixing at a high content (~ 4 wt%, even > 20 wt% [11]) is widely used, it often leads to increased system viscosity. This rise in viscosity can restrict subsequent manufacturing processes, such as the foaming of foam materials and the blow molding of films. Additionally, the introduction of bulk flame retardants can degrade tensile strength and elongation, making it challenging to achieve ideal low-density foams or highly flexible films [12]. Moreover, natural materials like wood products struggle to achieve effective flame retardance through above method. In contrast, surface fire-retardant coatings offer a promising means of providing robust fire protection for various substrates, including wood products, fabric, steel, polymer foams, and more [13–20]. Since these coatings localized on the surface, they have minimal impact on the mechanical or processing performance of the substrate. Typically, they form a thermostable fire-protective layer that acts as a physical barrier against mass and heat transfer when exposed to flame resource [21–23], thus preventing damage to the underlying materials. For this reason, some impressive efforts have been made to achieve a fire safety, especially combining nano-/microscale filler (e.g., nanocellulose, MXene, LDHs, graphene oxide (GO)) with adhesive macromolecule to create a composite coating [16, 22, 24–26]. For instance, Zhou et al. [27] reported a fire-retardant-coated polyurethane (PU) foam with a Na-montmorillonite (MMT) and graphitized multi-armed carbon nanotubes (GMWCNT) nanostructure using a layer-by-layer assembly, which exhibited improved nonflammability and reduced total smoke release (TSR). Similarly, a lava-inspired ceramifiable nanocoating (~ 200 μm) was reported, resulting in a rapidly self-extinguishing flame-PU foam [22]. Recently, our group [28] also reported an anti-corrosion coating with outstanding fire protection, leveraging the barrier effect of MXene nanosheets along with the heat absorption and gas dilution properties of LDHs. Despite these promising advances, existing flame-retardant coating still faces several challenges, including high production cost (e.g., MXene, GO), complex processing technology (e.g., plasma treatment), environmental friendliness of the processing process, unsatisfactory effectiveness (requiring thick coating), and visible light opacity (important for the aesthetic aspect wooden buildings). Therefore, developing reliable fire-retardant transparent coatings through simple and environmentally friendly processes remains a significant challenge.

Leveraging the synergistic effects between LDHs and boric acid [9], we have designed a transparent, ceramifiable, and fire-proof coating through the establishment of hierarchical hydrogen bonding networks within a green and environmentally friendly water-based system. Our research indicates that employing a specific material ratio fosters the creation of a ceramic-like and porous protective layer upon exposure to flame or heat source, thereby enhancing fire safety significantly. Our coating, with a low thickness of ~ 100 μm—thinner than most existing solutions—enables the coated wood to self-extinguish ability rapidly (within 2 s), achieve UL-94 V-0 rating, attain a high limiting oxygen index (LOI) of 37.3%, and maintain excellent optical transparency (85%), outperforming to most of previous counterparts. Furthermore, it preserves low conventional toxicity index (CIT_G_) value without significant amounts of harmful gases. This work paves the way for the environmentally friendly development of transparent fire-retardant coatings, offering substantial potential for applications in construction, transportation, and railway sectors.

## Experimental Section

### Materials

Sodium *p*-styrene sulfonate (SSS), 2-hydroxygenthyl methacrylate (HEMA), melamine (C_3_H_6_N_6_, MA), boric acid (H_3_BO_3_, BA), and ammonium persulfate ((NH_4_)_2_S_2_O_8_, APS) were obtained from China National Pharmaceutical Group Chemical Reagent Co., Ltd, China. Magnesium nitrate hexahydrate and aluminum nitrate nonahydrate were purchased from Xilong Science Co., Ltd, China. Silane surface treatment agent was synthesized in the laboratory. PU was acquired from Wuxi Kezhao Polyurethane Material, China. Other chemicals were used without further purification. Wood (pine) was supplied by Xiangfa Timber Processing Factory, China.

### Sample Preparation

#### Synthesis of LDH Nanosheets

Mg–Al LDH nanosheets with NO_3_^−^ were synthesized via a coprecipitation method. Mg(NO_3_)_2_⋅6H_2_O (0.3 M) and Al(NO_3_)_3_⋅9(H_2_O) (0.3 M) were added to vigorously stirred deionized water (DI) to form aqueous solution A. Meanwhile, the aqueous solution B, containing NaHCO_3_ (0.2 M) and NaOH (0.4 M), was prepared. Solution B was then gradually added to the solution A while maintaining the pH at approximately ~ 9.5, as monitored by a pH meter. The resulting sol–gel solution was subjected to hydrothermal treatment at 140 °C for 24 h, followed by washing with centrifugation and ultrasound three times. After drying, the white powder was obtained.

#### Synthesis of Poly(SSS-co-HEMA)

The addition polymerization of SSS and HEMA was conducted in a 250-mL three-neck round-bottom flask with mechanical stirring. For instance, to synthesize the poly(SSS_x_-*co*-HEMA_y_) (PSH) copolymer (x/y represents the molar ratio of SSS and HEMA): First, SSS, HEMA, APS, and deionized water were added to the 250-mL flask container, and the mixture was stirred to form a uniform solution while being heated 65 °C. After 3 h of stirring, additional APS (0.01 g) was subsequently added to the flake, and the reaction was continued at 65 °C for another 4 h. To remove the residual monomer, the poly(SSS-*co*-HEMA) aqueous solution was subjected to dialysis and then dried. Finally, for convenience, the copolymers, such as poly(SSS_1_-*co*-HEMA_2_), poly(SSS_1_-*co*-HEMA_1_), and poly(SSS_3_-*co*-HEMA_1_), were, respectively, named as PSH-1, PSH-2, and PSH-3.

#### Preparation of Melamine Diborate (M⋅2B)

The M⋅2B supramolecular was synthesized based on preciously reported works. Typically, MA and BA, with a molar ratio of 1:2, were dissolved in deionized water at 60 °C under stirring. After 1 h, a transparent solution was obtained.

#### Fabrication of Various Films and Coating Materials

Various PSH-based nanocomposite films were prepared following the hydrothermal and dry processes described in Table S2. First, a specific amount of BM (~ 0.06 g), LDHs (0.1 g), or BM/LDHs (0.16 g) aqueous was added to the PSH aqueous solution (15 wt%, ~ 0.780 g) and stirred magnetically for 4 h to form a uniform mixture. Next, the different PSH-based solutions were dried in an oven at 40 °C for approximately 10 h. Finally, various types of film samples with ~ 5 wt% coacting were fabricated after the drying process. The pure PSH film was obtained by directly drying the solution in an oven at 40 °C. For convenience, the films, e.g., PSH/BM, PSH/LDHs, and PSH/BM/LDHs, were named as PBM, PL, and PBML, respectively. To obtain the coating composite materials, the various PSH-based solutions were brushed onto prewashed and dried neat PU or wood surfaces. The resulting samples were then dried at 60 °C for 4 h to produce the modified flame-retardant PU foam or wood materials. Notable, the thickness of all coatings is controlled within 100 μm.

### Characterizations

The detailed characterization methods are provided in Supporting Information.

## Results and Discussion

### Design and Preparation of Transparency Nanosystem Coatings

**Fig. 1:**
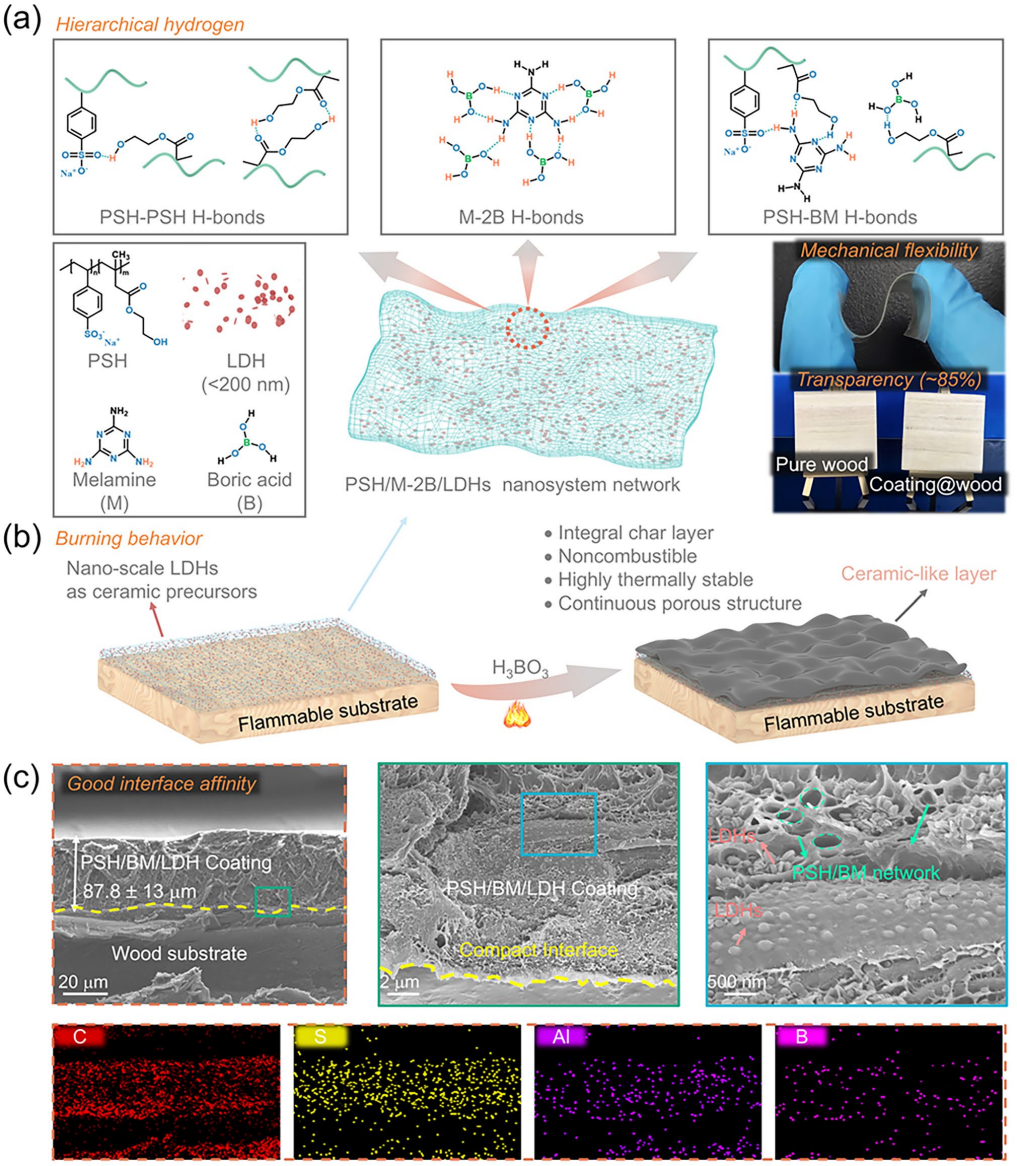
Design and fabrication of transparent ceramifiable fire-proof coating. **a** Schematic diagram of the hierarchical interactions between PSH chains and the supramolecular molecules in PSH/BM/LDH nanosystem network, showing mechanical flexibility and high transparency. **b** Schematic diagram for the mode of actions for the ceramic hybrid coating. **c** SEM images for the cross section morphology of the PSH/BM/LDH-coated wood along with EDS images (e.g., C, S, Al, and B)

To design a transparent ceramizable fire-retardant coating, three main building blocks are typically required. (I) A fire-proof polymer, which serves as the coating base as a glue, can easily form numerous chars with high thermal stability upon exposure to flame or a heat flux. The PSH was synthesized via a facile radical copolymerization of HEMA and SSS molecules in different ratios in a water medium (Table S1). The structure of copolymer was confirmed through proton nuclear magnetic resonance (^1^H NMR) (Fig. S1a) and Fourier-transferred infrared spectroscopic (FTIR) analyses (Fig. S1b). Generally, a higher SSS content or SSS/HEMA ratio results in better thermal stability and flame retardancy but weaker adhesion (Fig. S1c–e). (II) The two-dimensional layered-structural MgAl-LDHs, containing ceramic elements (e.g., Mg and Al), serve as a physical barrier that reinforces the char integrity [29, 30]. Additionally, LDHs contribute to heat absorption and gas dilution [9]. The nanoscale MgAl-LDHs were prepared using a classic in situ hydrothermal method, resulting in a size of approximately 135 nm (Fig. S2a, b, d). The structure was verified using X-ray diffraction (XRD) analysis (Fig. S2c, d). These LDHs integrate into the carbon layer, forming a more stable protective layer that further enhances flame-retardant properties. (III) The supermolecule M⋅2B not only acts as a binder, promoting the formation of a ceramic-like MgO or Al_2_O_3_-based coating from the LDHs, but also serves as an effective physical crosslinker to enhance the mechanical properties. It interacts with PSH molecules and LDHs to form a cross-linking nanosystem via amine, hydroxyl, and sulfonate groups, creating multiple hydrogen bonds within the composites (Fig. 1a) [26, 31, 32]. Interestingly, the combination of three components—PSH, nanoscale LDHs, and the supermolecule M⋅2B—enables the construction of a mechanically flexible, transparent, and self-extinguish film/coating, which is expected to produce a vitreous phase through the collaborative interaction of PSH, LDHs, and M⋅2B when placed on fire/heat source (more details later).

In addition, to balance the transparency and fire-proof properties of the material, we optimized the composition ratio of each component in the PBML film/coating (Table S2). To visually observe the microstructure of the composite coating on the wood substrate, we conducted the scanning electron microscope (SEM) to examine the cross section of as-designed PSH/BM/LDH@wood. Clearly, the coating layer with a Coral Reef structure shows a good interface compatibility with the wood, as evidenced by the continuous and tight connection between them (Fig. 1c, the top). More importantly, with a coating thickness of only ~ 87.8 μm, the wood achieves excellent fire-retardant properties (further evidence provided later). Both SEM and energy-dispersive X-ray (EDS) elemental mapping confirm the uniform distribution of the Mg and Al elements from LDH throughout the entire coating (Fig. 1c, bottom, and Fig. S3). The LDH nanosheets (marked by red arrows) are embedded in the polymeric network (marked by green arrows) (Fig. 1c, the right). Additionally, the elements B and P, originating from the supermolecule M⋅2B, are uniformly distributed in the top layer, highlighting the homogeneous dispersion of M⋅2B within the nanocomposite coating. In brief, a flame-retardant organic–inorganic nanosystem coating has been successfully constructed on the wood surface.

### Synthesis, Characterization, and Internal Interactions in PSH/BM/LDHs Coating

**Fig. 2:**
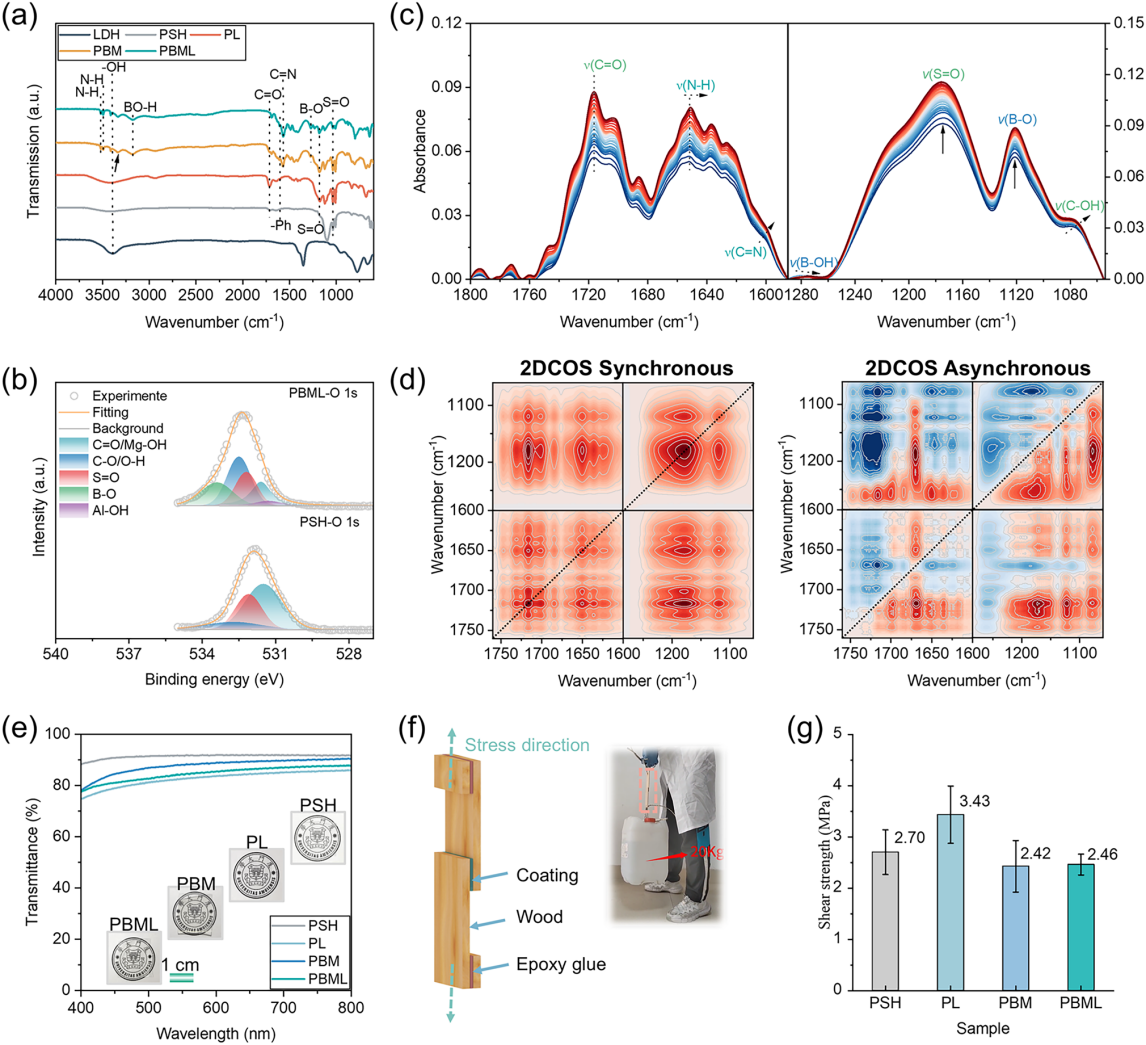
Structure characterization and interaction analysis in PSH/BM/LDHs. **a** FTIR spectra and assignments of LDH. PSH, PL. PBM, and PBML. **b** O 1*s* XPS spectra of PSH paper and PBML paper. **c** Temperature-variable FTIR spectra of PSH/BM/LDHs films upon heating from 25 to 55 °C in the regions of (interval: 1 °C). **d** 2DCOS synchronous and asynchronous spectra generated from (**c**). In the 2DCOS spectra, the red colors represent positive intensities, while green colors represent negative intensities. **e** UV–Vis transmission spectra of PSH film, PL film, PBM film, and PBML film. **f** The chart illustrating the adhesion or shear strength tests for wood. **g** Shear strength of various coating samples for wood

To validate the internal interactions within the representative PSH/BM/LDHs film, ATR-FTIR spectra of LDHs, PSH, PL, PBM, and PBML were compared, as shown in Fig. 2a. The five distinct peaks at 3493 cm^−1^ (N–H asymmetric stretching), 3412 cm^−1^ (N–H symmetric stretching), 3176 cm^−1^ (B-OH stretching), 1566 cm^−1^ (C=N stretching belonging to the melamine triazine ring), and 1270 cm^−1^ (B-OH stretching) in the spectra of PBML (and PBM) are associated with the supermolecule M⋅2B [33–35]. After introducing BM into the PSH or PL system, the *v*(-OH) and* v*(C=O) peaks shift to lower wavenumbers, while the *v*(C=O) peak of PL composite remains almost unchanged, indicating the formation of multiple hydrogen bonds between supermolecule M⋅2B and PSH molecules. Figure S4 presents the XPS results of the PSH film and PBML film to further confirm the chemical and elemental state composition of the PSH/BM/LDH composite. Compared to O 1*s* spectrum of pure PSH paper (Fig. 2b), the peaks of C–O/O–H, S=O, and C=O groups in the PBML film shift from 532.7, 532.1, and 531.5 eV to 532.5, 532.2, and 531.6 eV, respectively, further demonstrating the presence of multiple H-bonding [36].

Temperature-variable FTIR spectra of the as-prepared PSH/BM/LDHs film were recorded from 25 to 55 °C to further investigate the thermosensitivities and subtle changes of specified groups at the molecular level. As shown in Figs. 2c and S5, there is almost no wavenumber shift of the N–H and B-O groups upon heating. In contrast, with increase in temperature, the *v*(N–H), *v*(C=N), *v*(B-OH), and *v*(C–OH) peaks shift to lower wavenumbers, suggesting that the temperature increase enhances the association of these groups (e.g., N–H, B-OH, C=N, and C–OH). Two-dimensional correlation spectroscopy (2DCOS) was generated from the temperature-variable FTIR spectra, providing comprehensive information at molecular level [37–39]. Based on Noda’s judging rule, derived from the signs of cross-peaks in 2DCOS synchronous and asynchronous spectra (Fig. 2d and operation details in Table S3), the order is as follows: v(C–OH) → v(B-O) → v(S=O) → v(B-OH) → v(N–H) → v(C=N) → v(C=O). This order reveals the presence of hierarchical hydrogen bonding interactions, with the most thermally sensitive peak (1077 cm^−1^) corresponding to the highest bonding strength attributed to the C–OH [40].

Having elucidated the hierarchical hydrogen bonding within the network configuration, we proceed to investigate how it affects the transparency and the mechanical properties of PBML coatings. Typically, the supermolecule M⋅2B would precipitate from solution as needle-like crystalline structures when the water in the system evaporates [26]. However, the as-prepared PBM films exhibit high transparency, with light transmission higher than 85% in the 400–800 nm range, due to the contributions of hierarchical hydrogen bonds between the -SO_3_ of PSH and the -NH_2_ of the supermolecule M⋅2B (Fig. 2e) [32]. In addition, the nanoscale Mg–Al LDHs, with a small planar size of ~ 135 nm, are smaller than the wavelength of light at 200 nm, which minimally interfere with the high transparency (> 80%) of the PBML films. We further conducted the typical strain–stress experiments to explore the relationship between mechanical performance and the presence of multiple physical bonds. Incorporating a small amount of M⋅2B into the PSH molecule network effectively improved the tensile strength of PSH/BM films from 35.31 to 37.45 MPa (Fig. S6a, b). Along with the increase in the tensile strength, the elongation at break also increases, and similar enhancement on toughness can also generate (Fig. S6c) [41]. We note that the introduction of LDHs as a filler into the polymer matrix resulted in a slight reduction in the overall mechanical properties (Fig. S6), which is inevitable. Figure 2f shows the char for illustrating the adhesion or sheer strength tests for wood. The shear strength for different coatings follows the order: PL (3.43 MPa) > PSH (2.70 MPa) > PBML (2.46 MPa) > PBM (2.40 MPa) (Fig. 2g). The decrease in adhesive strength for PBML and PBM is largely attributed to the formation of multiple hydrogen bonds within the PBML coating system, which weakens the physical interactions with the abundant hydroxyl groups on the wood surface. Despite the lower sheer strength of the PBML coating (2.46 MPa) compared to pure PSH, as-designed PSH/BM/LDHs coating can support a 20-kg bucket of water (Fig. 2f inset, and Movie S1) and is comparable to most adhesive materials reported previously [22]. The high adhesion to wood is largely attributed to the good permeability of the coatings (Fig. S7).

### Validation of the Creation for Ceramic-Like Fire-Retardant Protective Layer

**Fig. 3:**
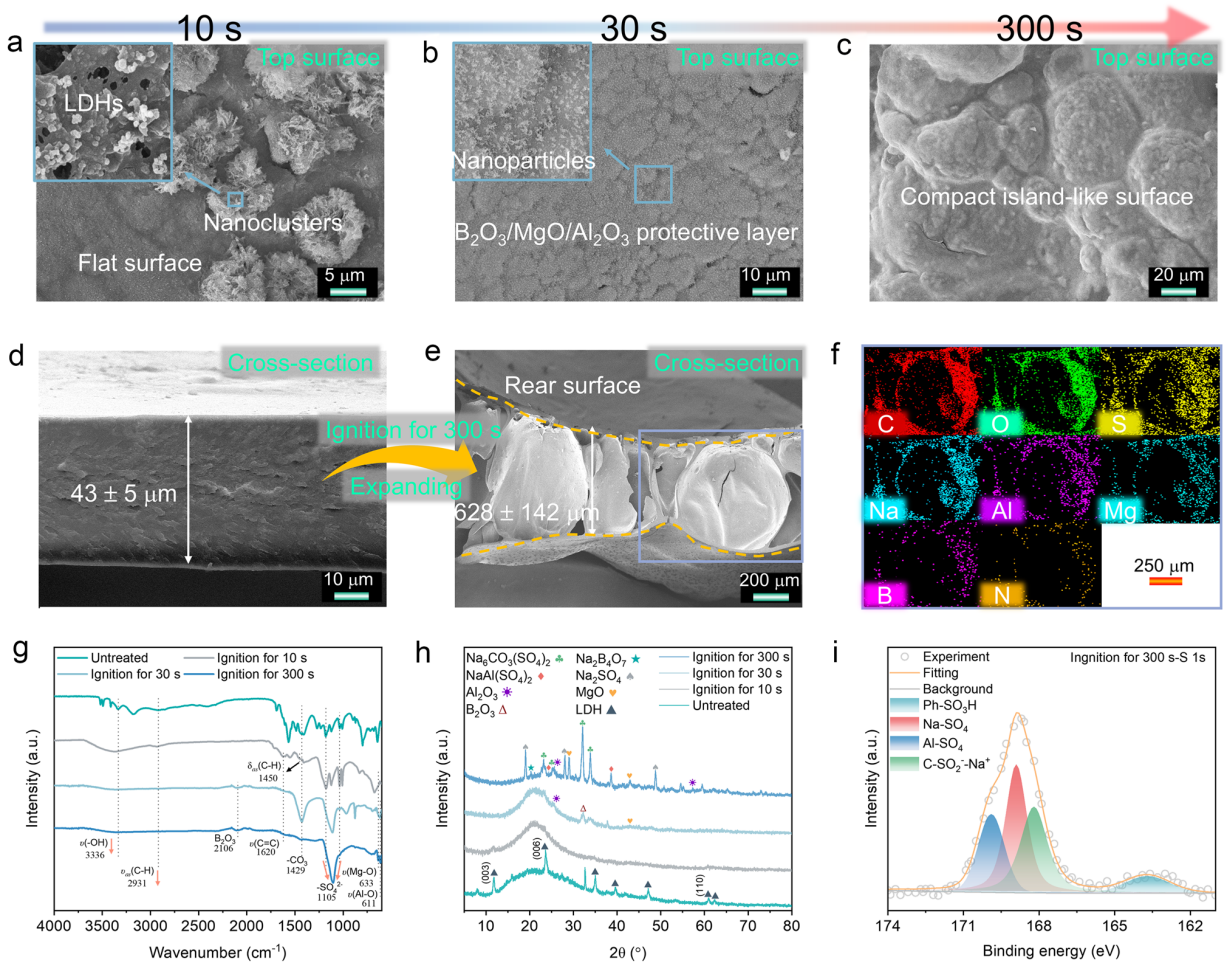
Formation mechanism of porous ceramic-like fire-proof protection layer. SEM images (EDS mapping) of the PBML paper: **a–c** top surface after being exposed to the alcohol lamp flame (~ 500 °C) for different time, **d** cross section before burned, and **e**–**f** cross section after burned as well as corresponding EDS mapping. **g–i** FTIR, XRD, and XPS of the PBML paper after burned with different time

The transparent PBML film with a Coral Reef structure exhibits excellent charring capability, a high expansion ratio, and superior flame-retardant properties after 11 s of ignition, without any droplet formation (Fig. S8 and Movie S5). To better distinguish the differences between various films after flame attack exposure, the microstructure and morphology of different PSH-based films were characterized by SEM, as shown in Fig. S9a_1_-d_1_. Clearly, compared with the damaged surface of pure PSH, PL, and PBM films, which exhibit obvious microporous structures after burning (Fig. S9a–c, Movies S2–S5), the burned PSH/BM/LDHs film shows an integrated and compact surface morphology without microcracks (Fig. S9d_2_-d_3_). This improvement is attributed to the reinforcing effect of boric acid. These results highlight that the combination of LDHs and M⋅2B contributes to the generation of a more integral and compact char layer, which is expected to offer better fire protection than binary hybrid coatings. Interestingly, some nanoclusters composed of LDHs remain on the surface exposed to flames for 10 s (see Figs. 3a and S9d_4_). When flame exposure time is extended to 30 s, the hybrid film gradually yields char residues, as visually evidenced by a coating color change from milky yellow to black (Fig. S10). In addition, the LDHs decompose completely into Al_2_O_3_/MgO mixture and H_2_O, leading to transformation of nanoclusters into nanoparticles (Fig. 3b) [9]. As the burning time increases to 300 s, the nanoparticles almost fully melt into the carbon layer, forming a new, compact, and integral ceramic-like layer, as indicated by the char color turning off-white (Fig. 3c). Moreover, the thermal degradation of PSH and LDHs releases abundant inert gas and promotes the formation of an internal foamed or porous structure within the char layer. As shown in Fig. S11a_2_-c_3_, many irregular pores and cavities ranging from several microns to hundreds of microns in size are clearly visible in the cross-sectional area. All as-processed films expand more than 16 times more than their original thickness (Fig. 3d, e), which is advantageous for insulating heat and flames. It is noteworthy that the size of the film’s pores becomes more uniform over time, indicating that further combustion of PBML gradually fills these pores in the outer layer. As shown in Figs. 3f and S11a_4_-c_4_, the expanded protective layer still contains elements such as C, O, S, Na, Al, Mg, B, and N elements even after burning for 300 s, with Al and B are well-known ceramicizable elements. Therefore, we suppose that the off-white product is a ceramic-like structure.

To further validate the formation mechanism of the condensed phase, we examined the chemical composition of the top surface of the charring layer after burning for different durations using FTIR, XRD, XPS, and Raman spectroscopy (Figs. 3g–i and S12–S14). As predicted, the results for specimens with different treatment times show a gradual change, consistent with the SEM images discussed above. A new vibration peak at 1620 cm^−1^ and a peak at 283.87 eV (C=C) were observed (FTIR and XPS C 1*s* spectra), but most of the characteristic peaks of the original film were observed (Figs. 3g, S4b, and S14a). Similar to the original sample, the specimen still exhibited fluorescence interference after burning for 10 s (Fig. S12b), indicating that it retains a relatively complete PPS unit structure with a fluorescence effect. With extended processing time, the intensity of the absorption peak located at 3336 cm^−1^ (*v*O–H) visibly decreased, while the peaks of organic functional groups gradually transform into inorganic functional groups (e.g., –CO_3_^2−^, –SO_4_^2−^, and B_2_O_3_) [22, 42]. After burning for 300 s, new characteristic peaks at 633 cm^−1^ (*v*Mg–O) and 611 cm^−1^ (*v*Al–O) and others noncombustible groups replaced the flammable functional groups. Additionally, as shown in Fig. S14d-f, the peaks assigned to H_3_BO_3_ (193.2 eV) and B–O–C (190 eV) disappear after burning, while peaks of highly thermally stable ingredients, such as B-C, B_2_O_3_, BN, and Na_2_B_4_O_7_, become visible [43–45]. Meanwhile, as time goes by, the *I*_D_/*I*_G_ ratio and the C–C/C=C peak ratio of char residues decrease from 3.48 to 3.02 and from 2.41 to 1.24, respectively (Figs. S12c, d and S14b, c; Table S4), indicating that the degree of graphitization gradually increases in the char. This increase is conducive to forming a higher thermal stable protective layer [46]. Moreover, the char of the film treated for 10 s shows a broad peak at 2θ = 21.4° in the XRD pattern, differing from the original sample (Fig. 3h), indicative of its amorphous structure. With flame treatment extending to 30 s, new peaks appear at 25.6°, 32.3°, and 43.0°, corresponding to Al_2_O_3_, B_2_O_3_, MgO, respectively. The char after 300 s shows more characteristic peaks of inorganic crystals, such as Na_6_CO_3_(SO_4_)_2_, Na_2_B_4_O_7_, NaAl(SO_4_)_2_, and Na_2_SO_4_. In addition, the XPS S 1*s* and Na 1*s* spectra exhibit noncombustible compositions, such as Na-SO_4_ (168.9 eV and (1071.2 eV)), Al-SO_4_ (169.89 eV), Ph-SO_3_H (163.7 eV), C–SO_2_^−^–Na^+^ (168.2 eV), Na–O (1072.5 eV), and Na-CO_3_ (1071.5 eV) (Figs. 3i and S13b) [47]. These results demonstrate that the PBML film forms a ceramic-like structure during the burning process. In summary, the above results indicate that the Al and Mg elements within the film gradually migrate from the interior of the charring layer to the surface (the side in contact with the fire or heat flux) and become enriched there during the combustion process. This migration forms a noncombustible, integral, foamed ceramic-like char layer under fire conditions, providing robust fire and thermal protections for the underlying substrates.

### Pyrolysis Behavior and Flame-Retardant Mechanism

**Fig. 4:**
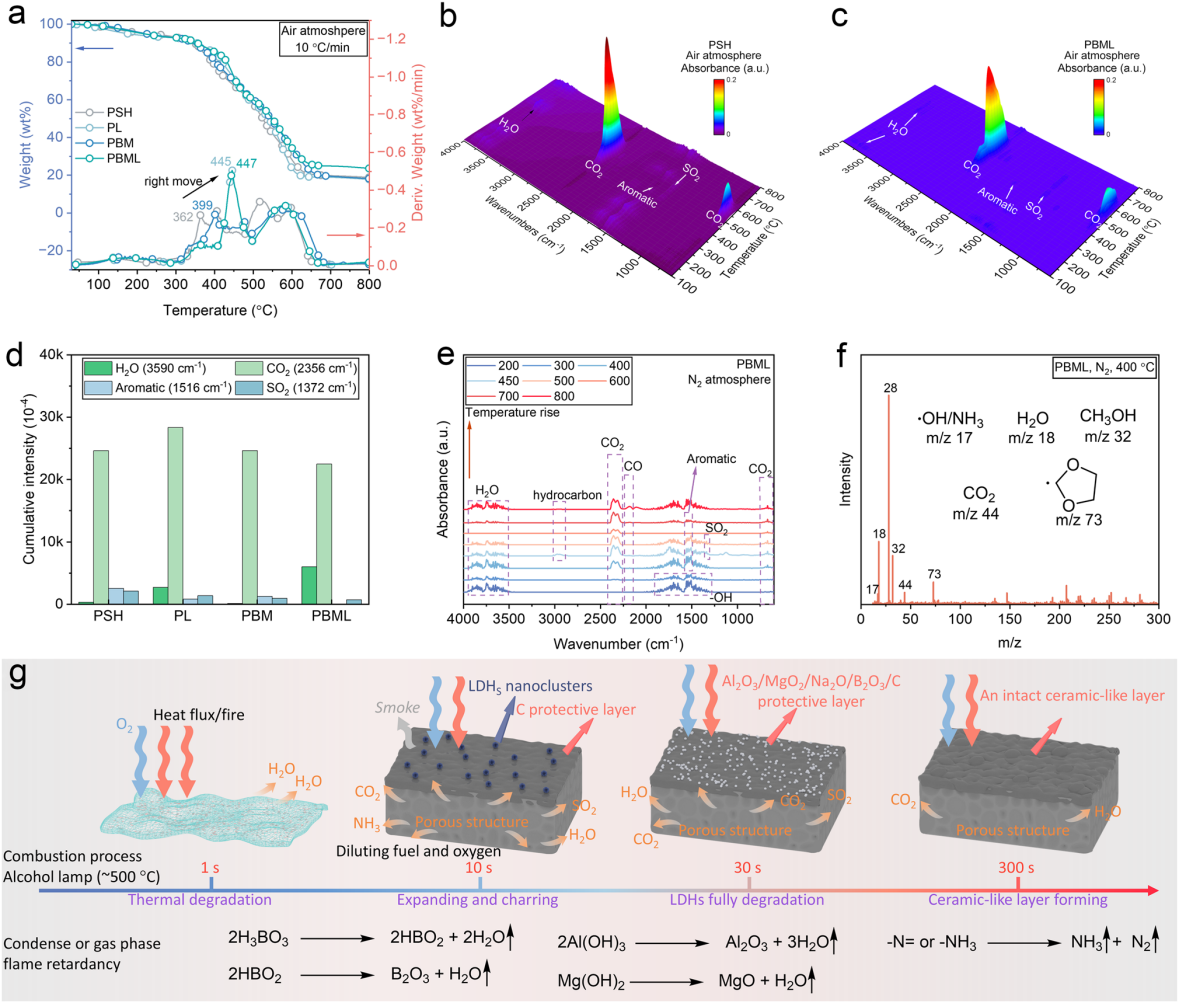
Pyrolysis behavior and flame-retardant mechanism of the PBML films. **a** TGA and DTG curves of PSH, PL, PBM, and PBML papers at air atmosphere. **b–c** The three-dimensional spectra of pyrolysis products of PSH and PBML paper under air atmosphere. **d** Gaseous phase products cumulative intensity under air atmosphere at 350–800 °C, and the cumulative intensity was obtained by integrating the normalized absorbance curve of PBML during 350–800 °C. **e** The infrared spectra of PBML under N_2_ atmosphere at various temperatures. **f** The pyrolysis products of PBML obtained from TGA coupled with GC–MS at 400 °C under N_2_ atmosphere. **g** Schematic illustration for the combustion as a function of flame treatment time for PBML film

The pyrolysis behaviors of different PSH-based samples were evaluated using thermogravimetric analysis (TGA). Under an air atmosphere, the TGA curves for PSH/M⋅2B/LDHs differ significantly from those of pure PSH molecules, as shown in Fig. 4a and Table S5. As observed in Fig. 4a, the thermal degradation process can be divided into two stages. The first stage involves the breakage of the soft HEMA segment at 237–327 °C, and the second stage entails the decomposition of the residual HEMA unit and the hard SSS segment at 332–671 °C (Fig. 4a) [48, 49]. As expected, most PSH-based composites exhibit a higher maximum weight loss temperature (*T*_max_ at 445, 399, and 447 °C, respectively) in comparison with pure PSH (362 °C), clearly suggesting an active role of LDHs or M⋅2B in enhancing the thermostability of the PSH matrix.

To further elucidate the flame-retardant mechanism in the gas phase of the hybrid materials, we utilized TGA coupled with FTIR (TGA-FTIR) and TGA coupled with GC–MS (TGA-GCMS) to determine the chemical compositions of volatile pyrolysis gaseous products. The analysis reveals that the main pyrolysis products of all samples under air atmosphere are CO_2_ (strong absorption peak at 2356 cm^−1^), H_2_O (3590 cm^−1^), SO_2_ (1372 cm^−1^), and aromatic compounds (1516 cm^−1^) (Figs. 4b, c and S15–S16), which primarily arise from PSH degradation. A quantitative analysis presented in Figs. 4d and S17 involves determining cumulative intensity by integrating the absorbance curve over the temperature interval from 100 to 800 °C (for CO_2_ and H_2_O) or 400–800 °C (for SO_2_ and aromatics; note: these two IR absorbance peaks are affected by water at low temperature) (Fig. S18). Remarkably, the cumulative intensity of aromatic and CO_2_ for PBML impregnated with LDHs and M⋅2B decreased to 29.8 × 10^–4^ and 22,457.5 × 10^–4^ a.u. g^−1^, respectively (Fig. 4d), implying that more aromatic molecules are contributing to the formation of the charring layer under the influence of both LDHs and M⋅2B. Moreover, we investigate the sample’s degradation under a N_2_ atmosphere to simulate the pyrolysis behavior of materials within the porous structure layer generated after combustion. The TGA results correspond with the regularity observed under air conditions (Fig. S19). However, all coating samples show more complex degradation products under N_2_ conditions, resulting in the formation of new species such as hydrocarbon (2943 cm^−1^), CO (2177 cm^−1^), ethylene (947 cm^−1^), and carbonyl compounds (1761 cm^−1^) (Figs. 4e and S20a_1_-d_2_) [48–50]. TGA-GCMS indicates that at around 400 °C, a significant amount of CO (m/z 28) is released, along with substantial amounts of H_2_O (m/z 18), CH_3_OH (m/z 32), and others organic fragments with larger m/z (> 70) (Fig. S20a_3_). In contrast, macromolecular fragments similar to the C_3_H_5_O_2_⋅ (m/z 73) significantly decline with the addition of LDHs or M⋅2B (Figs. 4f and S20b_3_-d_3_), further confirming their vital role in the conversion of the charring layer and suppression of combustible gas.

Given the aforementioned findings, the fire-retardant mechanism of the supramolecular nanosystem coating can be elucidated through three stages (Fig. 4g). In the initial stage (expose to fire for 1–10 s), the PSH polymer and LDHs begin to degrade, gradually creating a milky yellow porous layer with LDHs/C nanoclusters on the compact surface. This layer acts as a barrier, restricting the penetration of external O_2_ and heat flux/fire (condensed phase). Simultaneously, small volatile gas products are released, including noncombustible gases (e.g., CO_2_, SO_2_, NH_3_, and H_2_O) that inhibit the fire by diluting fuels and O_2_ (gas phase). As the burning time increases to 30 s, the upper BM and LDHs fully decompose into B_2_O_3_/Al_2_O_3_/MgO nanoparticles. These nanoparticles integrate into the charring layer, forming a compact, island-like (ceramic-like, vitreous phase) surface with an interior porous structure under the influence of boric acid at 300 s [9], maintaining long-time stability (> 300 s) during combustion. Overall, multiple fire-retardant mechanisms are produced in the PSH/MB/LDHs network, based on the formation of a ceramic-like protective layer and the release of nonflammable gases. This successfully endows the coatings with outstanding flame retardancy and high-temperature stability, effectively preventing the materials from igniting.

### Fire Safety of Ternary Hybrid Coating

**Fig. 5:**
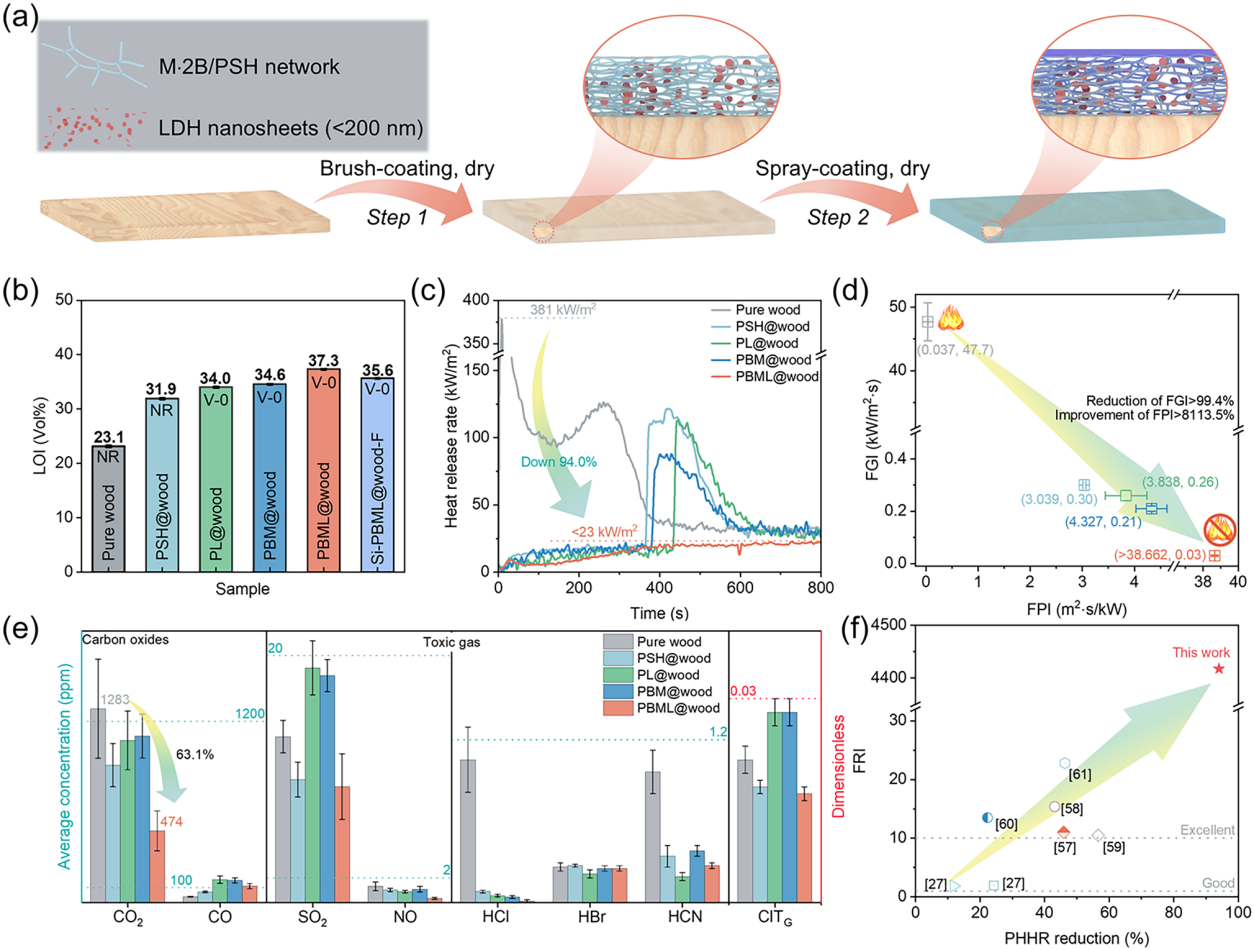
Coated wood exhibits enhanced fire-retardant. **a** Scheme for preparing the PSH/BM/LDH and Si-PSH/BM/LDH nanocoating on wood via brush-coating and spray-coating method. **b** LOI values and UL-94 rating for wood and coated wood. **c-d** HRR curves and FGI. **e** Concentrations of CO_2_, CO, SO_2_, NO, HCl, HBr, and HCN in the smoke toxicity test and general conventional index of toxicity (CIT_G_) obtained from smoke toxicity. **f** FRI value versus PHRR reduction

Owing to a combination of advantages, including good adhesion, high optical transparency, and outstanding high-temperature resistance, the PSH/BM/LDHs networks are well suited for use as a fire-proof coating for flammable wood and wood products. Herein, flame-retardant wood coated with a PSH/BM/LDHs coating was prepared using a brush-dry method (Fig. 5a). LOI tests and UL-94 vertical burning are standard bench-scale fire tests used to evaluate the ignitability and combustibility of materials in industrial setting. Uncoated wood exhibits a low LOI of 23.1% and does not achieve any UL-94 ratings, burning violently (> 134 s) and rapidly spreading flames until consumed once ignited (Figs. 5b and S21a; Movie S6). For PSH@wood, this achieves a higher LOI value of 31.9%, but unfortunately, it fails to reach the desired UL-94–0 rating, with a prolonged first burning time (> 117 s) and long charring lengths (> 10 cm) (Fig. S21b; Movie S7). Different from wood coated with PSH or binary hybrid coating (PSH/BM and PSH/LDHs), wood treated with the PSH/BM/LDHs nanocomposite coating attains an LOI as high as 37.3% and can self-extinguish within 2 s after each igniting (Fig. S21e), successfully achieving a satisfactory UL-94 V-0 rating (Fig. 5b; Movies S7–S10). These results further demonstrate the positive synergistic effect between BM and LDHs, which work cooperatively to generate a stronger ceramic-like layer.

We further assessed the fire safety of coated wood using cone calorimetry, a method that replicates real fire scenarios. At an incident heat flux of 50 kW m^−2^, the uncoated wood typically ignites rapidly, taking only 14 s to ignite when exposed to a fire source (Table S6). It reaches its first peak heat release rate (pHRR) of 381 kW m^−2^ at 14 s and the second pHHR at 125 s (Fig. 5c). In stark contrast, the other four specimens showed significantly prolonged time to ignition (t_ign_), ranging from 378 to 392 s, reduced total heat release (THR) and pHRR, and increased residue mass (Fig. S22a, b), indicating the generation of more residues during burning. Notably, the wood coated with PBML could not be ignited during the cone calorimetry test (Movie S11), which is attributed to the generation of a ceramic-like MgO or Al_2_O_3_-based porous protective layer, superior fire-retardant performance compared to other samples (Fig. S23). Additionally, we calculated the fire growth index (FGI) and the fire performance index (FPI) for these samples. In brief, higher FPI values and lower FGI values correspond to lower ignitability and fire risk of materials [22, 51]. As shown in Fig. 5d, all coated wood samples exhibited a 99.4% reduction in FGI and an 8113.5% increase in FPI, effectively preventing the risk of fire propagation at an early stage. Although the total smoke production (TSP) observed in wood coated with the hydride coating was higher than in pure wood, the time to peak smoke production rate (t_pSPR_) was delayed to 200 s or more (Fig. S22c, d), which is attributed to the smoldering combustion of coated wood, releasing more smoke in the later stages [52]. Notably, during the combustion process, PBML@wood exhibited a decrease in CO_2_ emission (from 1808 to 541 kg kg^−1^) and an increase in CO emission (from 108 to 174 kg kg^−1^) compared to the other four samples, attributed to the porous ceramic-like protective layer isolating the flame and air (Fig. S22e). This isolation results in incomplete combustion, which could increase smoke production [53]. Normally, smoke and toxic gases are the main causes of casualties in a fire accidents [51]. To precisely determine the toxicology of gases and the concentration of various gases released at each moment during a real fire scenario, FTIR couple with a cone calorimeter was employed (CC-FTIR) following EN 45545–2/ISO 5659–2 standards [54–56]. Figure 5e clearly illustrates that during combustion, the CO_2_ and toxic gases levels were significantly lower than those of other samples. The presence of the isolation layer not only reduced the CO_2_ concentration by 63.1%, but also diminished the levels of toxic gases (such as SO_2_, NO_x_, HF, HCl, HCN, and HBr), as indicated in Table S7. In general, all toxic smoke gases are below the immediately dangerous to life and healthy concentration (Table S8) [56]. The conventional toxicity index (CIT_G_) of all composites in this study was lowest value for PBML@wood at 0.016, demonstrating the excellent smoke toxicity inhibitory effect of the ternary nanohybrid system coating.

Additionally, we compare the flame-retardant properties of our as-prepared PSH/BM/LDHs coating composite with other reported similar coated composites material systems, and the results demonstrate the superiority of the PBML@wood materials (Table S9). Overall, the PSH/BM/LDHs@wood (FRI > 4417.4 under a heat flux of 50 kW m^−2^) exhibited a greater increase in FRI than previous counterparts (Fig. 5f) and a significant reduction in pHRR compared to most previously coated wood materials [27, 57–61]. In comparison with other existing other fire-proof coated-substrate, such as PU [22, 46, 62, 63], SiRF [15], and cotton [64], our nanocoating leads to a higher LOI value while maintaining better transparency (~ 85%). In summary, our PSH/BM/LDHs hybrid coating excels in improving the LOI value and reducing the pHRR of wood, underscoring its intrinsic advantages over existing fire-retardant coating.

### High-Temperature Ablation and Other Application

**Fig. 6:**
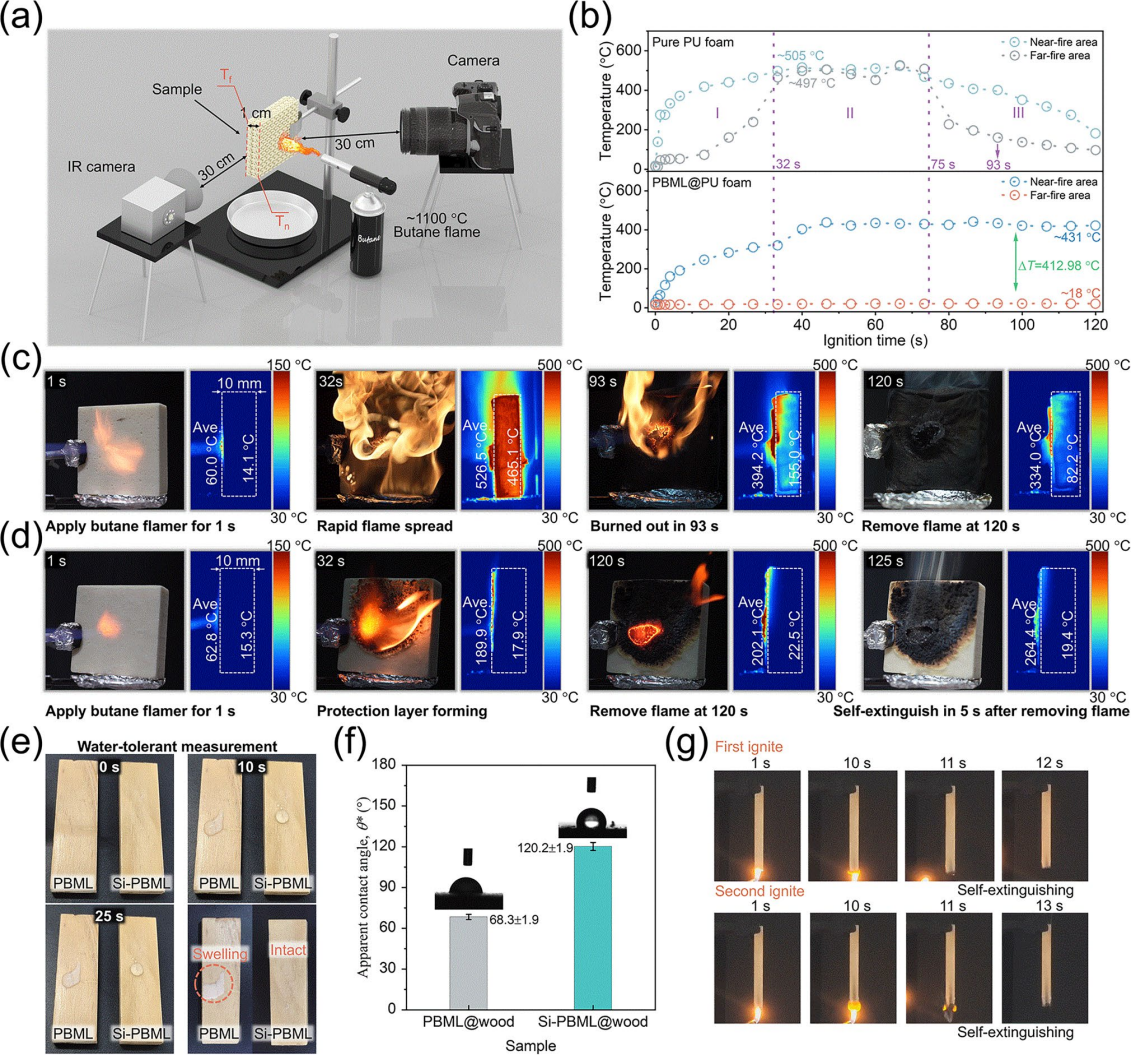
The nanosystem coating offers desired fire protections for PU foam. **a** The homemade setup for assessing fire behaviors. **b** Average temperature on T_n_ and T_f_ of the side surface PU foam as a function of burning time. **c** PU and **d** PBML@PU under the butane flame for 120 s, along with their side temperature variation with time determined by the IR camera. **e** Water-tolerant test, **f** the apparent contact angle θ*, and** g** UL-94 test of PBML-coated wood and Si-PBML-coated wood

Notably, there are many inorganic phases similar to those observed in the sample after flame treatment for 300 s when the temperature is set to above 500 °C (Figs. S24 and S25, with more details described in Supplementary information). This indicates that higher temperatures are conducive to the formation of a ceramic-like protective layer, providing better fire protection. Therefore, we constructed a homemade fire-testing setup to visually evaluate the fire protection capability of as-designed nanosystem coating for PU foam (or wood) by monitoring flame spread and recording the variation in side surface temperature under the attack of a butane flame (reaching temperatures as high as ~ 1100 °C) (Fig. 6a). Upon exposure to the flame, untreated PU foam ignited within 1 s (Fig. 6b, c; Movies S12 and S13) due to its organic composition and porous structure. The combustion process of PU foam can be summarized into three stages: (I) the flame propagation (0 ~ 32 s), (II) intense burning (32 ~ 75 s), and (III) depletion of fuel (75 ~ 120 s). By 93 s, the foam was completely burned out, leaving a deformed char with reduced dimensions (Fig. 6c). With trinary PSH/BM/LDHs hybrid coating, the coated PU foam can still ignite upon contact with flame source, but a nonflammable protective layer is generated, restraining fire or heat flux transmission (Fig. 6d; Movies S14 and S15). Notably, the average temperature at the far-fire area (L_f_) (~ 18 °C) is significantly lower than the temperature at the near-fire area (L_n_) temperature (~ 431 °C) and the L_f_ temperature of uncoated PU within 40–120 s (Fig. 6b; Movies S13 and S15). In addition, the PBML@PU self-extinguishes in 5 s of flame removal, maintaining an integral structure far superior to that of the uncoated (Figs. 6d-right and S27a). It should be noted that a similar experimental setup with untreated and coated wood also yielded comparable results (Figs. S26 and S27b; Movies S16–S19). Due to its exceptional fire protection at high temperature, the nanosystem composite coatings could serve as an ideal fire protection layer in high-demand scenarios. Finally, to mitigate the moisture sensitivity of the nanocoating, the coating surface was further treated to create a thin hydrophobic layer using a silane prepolymer.

As expected, compared to PBML@wood, the surface of Si-PBML@wood maintains an intact morphology, and its water contact angle is as high as 120.2°, significantly higher than 68.3° of the unmodified coated sample (Fig. 6e, f; Movie S20). Moreover, this coated wood still exhibits a high LOI of 35.6% and achieves the desired UL-94 V-0 rating (Figs. 5b and 6g; Movie S21). Therefore, the hybrid coating, which combines ceramifiable PBML coating and Si-coating, shows great potential for fire protections for wooden structures in buildings and forests in the future.

## Conclusions

In summary, inspired by ceramic materials, we have designed a nanoscale transparent fire-proof coating by combing nano-LDHs, the supermolecule M⋅2B, and a nonflammable PSH macromolecule. This unique coating, approximately 87.8 μm thick, enables wood to self-extinguish fires at an exceptionally early stage, significantly reduces heat release, achieves a high LOI value of 37.3%, and secures a desired UL-94 V-0 rating. Our research has uncovered that this organic–inorganic hybrid coating can form a more efficient, porous, flame-retardant protective layer with a vitreous phase due to the cooperative work between PSH, LDHs, and the M⋅2B as a time function of fire attack. The resulting char layer acts as a fire/thermal shield, providing effective fire protection for wood or PU foam. Furthermore, the CIT_G_ the PBML@wood coated with the silane prepolymer exhibits hydrophobic and flame retardancy. Since each component used is cost-effective and readily available, and the process aligns with the principles of green engineering, this composite material holds immense promise for widespread application. Given the prevalence of flammable materials in the modern society, we anticipate that this work could lead to significant improvements in fire safety in the fields of building and construction, as well as transportation.

## Supplementary Information

Below is the link to the electronic supplementary material.

Supplementary file1 (MP4 2275 KB)

Supplementary file2 (MP4 23940 KB)

Supplementary file3 (MP4 26865 KB)

Supplementary file4 (MP4 25350 KB)

Supplementary file5 (MP4 27548 KB)

Supplementary file6 (MP4 9466 KB)

Supplementary file7 (MP4 9811 KB)

Supplementary file8 (MP4 4687 KB)

Supplementary file9 (MP4 4460 KB)

Supplementary file10 (MP4 6947 KB)

Supplementary file11 (MP4 9431 KB)

Supplementary file12 (MP4 7735 KB)

Supplementary file13 (MP4 8947 KB)

Supplementary file14 (MP4 5182 KB)

Supplementary file15 (MP4 3912 KB)

Supplementary file16 (MP4 2918 KB)

Supplementary file17 (MP4 3736 KB)

Supplementary file18 (MP4 2896 KB)

Supplementary file19 (MP4 3609 KB)

Supplementary file20 (MP4 9947 KB)

Supplementary file21 (MP4 8847 KB)

Supplementary file22 (DOCX 28620 KB)

## References

[1] D.M.J.S. Bowman, J.K. Balch, P. Artaxo, W.J. Bond, J.M. Carlson et al., Fire in the earth system. Science **324**(5926), 481–484 (2009). 10.1126/science.1163886 19390038 10.1126/science.1163886

[2] H. He, Y. Qin, Z. Zhu, Q. Jiang, S. Ouyang et al., Temperature-arousing self-powered fire warning e-textile based on p–n segment coaxial aerogel fibers for active fire protection in firefighting clothing. Nano-Micro Lett. **15**, 226 (2023). 10.1007/s40820023-01200-8 10.1007/s40820-023-01200-8 PMC10575845 37831274

[3] I. Džolev, M. Laban, S. Draganić, Survey based fire load assessment and impact analysis of fire load increment on fire development in contemporary dwellings. Saf. Sci. **135**, 105094 (2021). 10.1016/j.ssci.2020.105094

[4] Y. Gao, C. Huang, Y. Chen, X. Chen, Y. Shen et al., Sustainable production of in-situ CO_2_ capture and mineralization of multifunctional nanowood with excellent anisotropic, flame retardant, and durability for building construction. Ind. Crops Prod. **221**, 119353 (2024). 10.1016/j.indcrop.2024.119353

[5] Y. Praticò, J. Ochsendorf, S. Holzer, R.J. Flatt, Post-fire restoration of historic buildings and implications for Notre-Dame de Paris. Nat. Mater. **19**(8), 817–820 (2020). 10.1038/s41563-020-0748-y 32669627 10.1038/s41563-020-0748-y

[6] M. Aram, X. Zhang, D. Qi, Y. Ko, Scaling study of smoke spread from building integrated photovoltaic (BIPV) double skin façade fire for achieving sustainable buildings and cities. Sustain. Cities Soc. **97**, 104648 (2023). 10.1016/j.scs.2023.104648

[7] N.A.F.M.N. Ong, M.A. Sadiq, M.S.M. Said, G. Jomaas, M.Z.M. Tohir et al., Fault tree analysis of fires on rooftops with photovoltaic systems. J. Build. Eng. **46**, 103752 (2022). 10.1016/j.jobe.2021.103752

[8] M. Aram, X. Zhang, D. Qi, Y. Ko, A state-of-the-art review of fire safety of photovoltaic systems in buildings. J. Clean. Prod. **308**, 127239 (2021). 10.1016/j.jclepro.2021.127239

[9] Y. Gao, J. Wu, Q. Wang, C.A. Wilkie, D. O’Hare, Flame retardant polymer/layered double hydroxide nanocomposites. J. Mater. Chem. A **2**(29), 10996–11016 (2014). 10.1039/C4TA01030B

[10] Y. Xue, X. Zuo, L. Wang, Y. Zhou, Y. Pan et al., Enhanced flame retardancy of poly(lactic acid) with ultra-low loading of ammonium polyphosphate. Compos. B Eng. **196**, 108124 (2020). 10.1016/j.compositesb.2020.108124

[11] M. Kim, J. Kim, Enhancement of the flame retardant properties of PPS-based composites via the addition of melamine-coated CaAl-LDH fire-retardant filler. Eur. Polym. J. **201**, 112584 (2023). 10.1016/j.eurpolymj.2023.112584

[12] S.T. Lazar, T.J. Kolibaba, J.C. Grunlan, Flame-retardant surface treatments. Nat. Rev. Mater. **5**(4), 259–275 (2020). 10.1038/s41578-019-0164-6

[13] A.N. Zhang, H.B. Zhao, J.B. Cheng, M.E. Li, S.L. Li et al., Construction of durable eco-friendly biomass-based flame-retardant coating for cotton fabrics. Chem. Eng. J. **410**(15), 128361 (2021). 10.1016/j.cej.2020.128361

[14] D. Rodriguez-Melendez, N.A. Vest, T.J. Kolibaba, Y. Quan, Z. Zhang et al., Boron-based polyelectrolyte complex nanocoating for fire protection of engineered wood. Cellulose **31**(5), 3083–3094 (2024). 10.1007/s10570-024-05773-4

[15] H.-Y. Chen, Z.-Y. Chen, M. Mao, Y.-Y. Wu, F. Yang et al., Self-adhesive polydimethylsiloxane foam materials decorated with MXene/cellulose nanofiber interconnected network for versatile functionalities. Adv. Funct. Mater. **33**(48), 2304927 (2023). 10.1002/adfm.202304927

[16] X. Wang, Z. Lei, X. Ma, G. He, T. Xu et al., A lightweight MXene-Coated nonwoven fabric with excellent flame retardancy, EMI Shielding, and electrothermal/photothermal conversion for wearable heater. Chem. Eng. J. **430**, 132605 (2022). 10.1016/j.cej.2021.132605

[17] Y. Zhang, Y. Huang, M.-C. Li, S. Zhang, W. Zhou et al., Bioinspired, stable adhesive Ti_3_C_2_T_x_ MXene-based coatings towards fire warning, smoke suppression and VOCs removal smart wood. Chem. Eng. J. **452**, 139360 (2023). 10.1016/j.cej.2022.139360

[18] Y. Wang, Y. Cheng, C. Yin, J. Zhang, X. Zhang et al., Seashell-inspired switchable waterborne coatings with complete biodegradability, intrinsic flame-retardance, and high transparency. ACS Nano **17**(13), 12433–12444 (2023). 10.1021/acsnano.3c01866 37338121 10.1021/acsnano.3c01866

[19] L. Pezzato, S. Akbarzadeh, A.G. Settimi, E. Moschin, I. Moro et al., Corrosion and antifouling properties of copper-containing PEO coatings produced on steels. Surf. Coat. Technol. **482**, 130631 (2024). 10.1016/j.surfcoat.2024.130631

[20] H. Zhang, X. Bu, W. Li, M. Cui, X. Ji et al., A skin-inspired design integrating mechano–chemical–thermal robustness into superhydrophobic coatings. Adv. Mater. **34**(31), 2203792 (2022). 10.1002/adma.202203792 10.1002/adma.202203792 35687054

[21] B. Lin, A.C.Y. Yuen, S. Oliver, J. Liu, B. Yu et al., Dual functionalisation of polyurethane foam for unprecedented flame retardancy and antibacterial properties using layer-by-layer assembly of MXene chitosan with antibacterial metal particles. Compos. B Eng. **244**, 110147 (2022). 10.1016/j.compositesb.2022.110147

[22] Z.W. Ma, J.Z. Zhang, C. Maluk, Y.M. Yu, S.M. Seraji et al., A lava-inspired micro/nano-structured ceramifiable organic-inorganic hybrid fire-extinguishing coating. Matter **5**(3), 911–932 (2022). 10.1016/j.matt.2021.12.009

[23] Z. Ma, X. Liu, X. Xu, L. Liu, B. Yu et al., Bioinspired, highly adhesive, nanostructured polymeric coatings for superhydrophobic fire-extinguishing thermal insulation foam. ACS Nano **15**(7), 11667–11680 (2021). 10.1021/acsnano.1c02254 34170679 10.1021/acsnano.1c02254

[24] L. Wang, P.V. Kelly, N. Ozveren, X. Zhang, M. Korey et al., Multifunctional polymer composite coatings and adhesives by incorporating cellulose nanomaterials. Matter **6**(2), 344–372 (2023). 10.1016/j.matt.2022.11.024

[25] W. Cai, N. Hong, X. Feng, W. Zeng, Y. Shi et al., A facile strategy to simultaneously exfoliate and functionalize boron nitride nanosheets via Lewis acid-base interaction. Chem. Eng. J. **330**, 309–321 (2017). 10.1016/j.cej.2017.07.162

[26] C.F. Cao, B. Yu, J. Huang, X.L. Feng, L.Y. Lv et al., Biomimetic, mechanically strong supramolecular nanosystem enabling solvent resistance, reliable fire protection and ultralong fire warning. ACS Nano **16**(12), 20865–20876 (2022). 10.1021/acsnano.2c08368 36468754 10.1021/acsnano.2c08368

[27] X. Zhou, Q. Fu, Z. Zhang, Y. Fang, Y. Wang et al., Efficient flame-retardant hybrid coatings on wood plastic composites by layer-by-layer assembly. J. Clean. Prod. **321**, 128949 (2021). 10.1016/j.jclepro.2021.128949

[28] H. Liu, W. Xu, H. Ren, D. Li, J. He et al., Integrated comprehensive protection coating achieved by ligand engineering modulated MXene@LDH heterojunction with anti-corrosion, electromagnetic wave absorption and fire safety. Chem. Eng. J. **486**, 150444 (2024). 10.1016/j.cej.2024.150444

[29] G. Camino, A. Maffezzoli, M. Braglia, M. De Lazzaro, M. Zammarano, Effect of hydroxides and hydroxycarbonate structure on fire retardant effectiveness and mechanical properties in ethylene-vinyl acetate copolymer. Polym. Degrad. Stab. **74**(3), 457–464 (2001). 10.1016/S0141-3910(01)00167-7

[30] C.M. Becker, A.D. Gabbardo, F. Wypych, S.C. Amico, Mechanical and flame-retardant properties of epoxy/Mg–Al LDH composites. Compos. Part A Appl. Sci. Manuf. **42**(2), 196–202 (2011). 10.1016/j.compositesa.2010.11.005

[31] A. Roy, A. Choudhury, C.N.R. Rao, Supramolecular hydrogen-bonded structure of a 1:2 adduct of melamine with boric acid. J. Mol. Struct. **613**(1), 61–66 (2002). 10.1016/S0022-2860(02)00128-X

[32] A.S. Kazachenko, N. Issaoui, M. Medimagh, OYu. Fetisova, Y.D. Berezhnaya et al., Experimental and theoretical study of the sulfamic acid-urea deep eutectic solvent. J. Mol. Liq. **363**, 119859 (2022). 10.1016/j.molliq.2022.119859

[33] J. Schott, J. Kretzschmar, M. Acker, S. Eidner, M.U. Kumke et al., Formation of a Eu(III) borate solid species from a weak Eu(III) borate complex in aqueous solution. Dalton Trans. **43**(30), 11516–11528 (2014). 10.1039/C4DT00843J 24849080 10.1039/c4dt00843j

[34] L. Jun, X. Shuping, G. Shiyang, FT-IR and Raman spectroscopic study of hydrated borates. Spectrochim. Acta A Mol. Biomol. Spectrosc. **51**(4), 519–532 (1995). 10.1016/0584-8539(94)00183-C

[35] Z. Lixia, Y. Tao, W. Jiang, G. Shiyang, FT-IR and Raman spectroscopic study of hydrated rubidium (cesium) borates and alkali double borates. Russ. J. Inorg. Chem. **52**(11), 1786–1792 (2007). 10.1134/S0036023607110241

[36] C.F. Cao, B. Yu, B.F. Guo, W.J. Hu, F.N. Sun et al., Bio-inspired, sustainable and mechanically robust graphene oxide-based hybrid networks for efficient fire protection and warning. Chem. Eng. J. **439**, 140161 (2022). 10.1016/j.cej.2022.140161

[37] W. Zhang, B. Wu, S. Sun, P. Wu, Skin-like mechanoresponsive self-healing ionic elastomer from supramolecular zwitterionic network. Nat. Commun. **12**(1), 4082 (2021). 10.1038/s41467-021-24382-4 34215738 10.1038/s41467-021-24382-4 PMC8253733

[38] Y. Wang, S. Sun, P. Wu, Adaptive ionogel paint from room-temperature autonomous polymerization of *α*-thioctic acid for stretchable and healable electronics. Adv. Funct. Mater. **31**(24), 2101494 (2021). 10.1002/adfm.202101494

[39] R. Fritzsch, S. Hume, L. Minnes, M.J. Baker, G.A. Burley et al., Two-dimensional infrared spectroscopy: an emerging analytical tool? Analyst **145**(6), 2014–2024 (2020). 10.1039/C9AN02035G 32051976 10.1039/c9an02035g

[40] Z. Fang, H. Mu, Z. Sun, K. Zhang, A. Zhang et al., 3D printable elastomers with exceptional strength and toughness. Nature **631**, 783–788 (2024). 10.1038/s41586-024-07588-6 38961297 10.1038/s41586-024-07588-6

[41] L. Jin, W. Cao, P. Wang, N. Song, P. Ding, Interconnected MXene/graphene network constructed by soft template for multi-performance improvement of polymer composites. Nano-Micro Lett. **14**, 133 (2022). 10.1007/s40820-022-00877-7 10.1007/s40820-022-00877-7 PMC9198158 35699778

[42] A. Sommer, D. White, M.J. Linevsky, D.E. Mann, Infrared absorption spectra of B_2_O_3_, B_2_O_2_, and BO_2_ in solid argon matrices. J. Chem. Phys. **38**(1), 87–98 (1963). 10.1063/1.1733501

[43] J. Cui, Y. Yang, X. Li, W. Yuan, Y. Pei, Toward a slow-release borate inhibitor to control mild steel corrosion in simulated recirculating water. ACS Appl. Mater. Interfaces **10**(4), 4183–4197 (2018). 10.1021/acsami.7b15507 29294269 10.1021/acsami.7b15507

[44] S. Guan, M. Qi, C. Wang, S. Wang, W. Wang, Enhanced cytocompatibility of Ti_6_Al_4_V alloy through selective removal of Al and V from the hierarchical micro-arc oxidation coating. Appl. Surf. Sci. **541**, 148547 (2021). 10.1016/j.apsusc.2020.148547

[45] B.J. Matsoso, K. Ranganathan, B.K. Mutuma, T. Lerotholi, G. Jones et al., Synthesis and characterization of boron carbon oxynitride films with tunable composition using methane, boric acid and ammonia. New J. Chem. **41**(17), 9497–9504 (2017). 10.1039/C7NJ01886J

[46] Y.B. Huang, S.H. Jiang, R.C. Liang, P. Sun, Y. Hai et al., Thermal-triggered insulating fireproof layers: a novel fire-extinguishing MXene composites coating. Chem. Eng. J. **391**, 123621 (2020). 10.1016/j.cej.2019.123621

[47] N. Maiti, S. Thomas, A. Debnath, S. Kapoor, Raman and XPS study on the interaction of taurine with silver nanoparticles. RSC Adv. **6**(61), 56406–56411 (2016). 10.1039/C6RA09569K

[48] K. Demirelli, M. Coşkun, E. Kaya, A detailed study of thermal degradation of poly(2-hydroxyethyl methacrylate). Polym. Degrad. Stab. **72**(1), 75–80 (2001). 10.1016/S0141-3910(00)00204-4

[49] D.D. Jiang, Q. Yao, M.A. McKinney, C.A. Wilkie, TGA/FTIR studies on the thermal degradation of some polymeric sulfonic and phosphonic acids and their sodium salts. Polym. Degrad. Stab. **63**(3), 423–434 (1999). 10.1016/S0141-3910(98)00123-2

[50] C. Peniche-Covas, W. Argüelles-Monal, J.S. Román, A kinetic study of the thermal degradation of chitosan and a mercaptan derivative of chitosan. Polym. Degrad. Stab. **39**(1), 21–28 (1993). 10.1016/0141-3910(93)90120-8

[51] W. Luo, M.-J. Chen, T. Wang, J.-F. Feng, Z.-C. Fu et al., Catalytic polymer self-cleavage for CO_2_ generation before combustion empowers materials with fire safety. Nat. Commun. **15**(1), 2726 (2024). 10.1038/s41467-024-46756-0 38548723 10.1038/s41467-024-46756-0 PMC10978860

[52] J. Li, C. Zhu, Z. Zhao, P. Khalili, M. Clement et al., Fire properties of carbon fiber reinforced polymer improved by coating nonwoven flame retardant mat for aerospace application. J. Appl. Polym. Sci. **136**(30), 47801 (2019). 10.1002/app.47801

[53] E. Kandare, P. Luangtriratana, B.K. Kandola, Fire reaction properties of flax/epoxy laminates and their balsa-core sandwich composites with or without fire protection. Compos. B Eng. **56**, 602–610 (2014). 10.1016/j.compositesb.2013.08.090

[54] 45545–2 railway applications, Fire protection on railway vehicles-part 2: Requirements for fire behaviour of materials and components. EN 45545–2. 2016. https://pmpgroup.org/wp-content/uploads/2021/10/PMP-EN-45545-2-Railway-Standrad.pdf

[55] 5659–2, plastics--smoke generation-part 2: determination of optical density by a single-chamber test. ISO 5659–2:2017. 2006. https://www.iso.org/standard/65243.html

[56] C. Zhu, S. Li, J. Li, M. Clement, C. Rudd et al., Fire performance of sandwich composites with intumescent mat protection: evolving thermal insulation, post-fire performance and rail industry testing. Fire Saf. J. **116**, 103205 (2020). 10.1016/j.firesaf.2020.103205

[57] C. Deng, Y. Liu, H. Jian, Y. Liang, M. Wen et al., Study on the preparation of flame retardant plywood by intercalation of phosphorus and nitrogen flame retardants modified with Mg/Al-LDH. Constr. Build. Mater. **374**, 130939 (2023). 10.1016/j.conbuildmat.2023.130939

[58] T. Zhang, J. Xi, S. Qiu, B. Zhang, Z. Luo et al., Facilely produced highly adhered, low thermal conductivity and non-combustible coatings for fire safety. J. Colloid Interface Sci. **604**, 378–389 (2021). 10.1016/j.jcis.2021.06.135 34265692 10.1016/j.jcis.2021.06.135

[59] L. Ma, T. Zhang, Y. Zhao, T. Yuan, X. Wang et al., Preparation of multifunctional flame-retardant and superhydrophobic composite wood by iron ions doped phytic acid-based nanosheets. Constr. Build. Mater. **422**, 135854 (2024). 10.1016/j.conbuildmat.2024.135854

[60] Q. Liu, H. Luo, Z. Gao, Y. Huang, J. Liang et al., Preparation of waterborne intumescent flame-retardant coatings using adenosine-based phosphonates for wood surfaces. Prog. Org. Coat. **187**, 108061 (2024). 10.1016/j.porgcoat.2023.108061

[61] F. Song, T. Liu, Q. Fan, D. Li, R. Ou et al., Sustainable, high-performance, flame-retardant waterborne wood coatings via phytic acid based green curing agent for melamine-urea-formaldehyde resin. Prog. Org. Coat. **162**, 106597 (2022). 10.1016/j.porgcoat.2021.106597

[62] D. Jiao, H. Sima, X. Shi, C. Zhang, B. Liu, Mussel-inspired flame retardant coating on polyurethane foam. Chem. Eng. J. **474**, 145588 (2023). 10.1016/j.cej.2023.145588

[63] H. Kim, D.W. Kim, V. Vasagar, H. Ha, S. Nazarenko et al., Polydopamine-graphene oxide flame retardant nanocoatings applied via an aqueous liquid crystalline scaffold. Adv. Funct. Mater. **28**(39), 1803172 (2018). 10.1002/adfm.201803172

[64] J. Xu, Y. Niu, Z. Xie, F. Liang, F. Guo et al., Synergistic flame retardant effect of carbon nanohorns and ammonium polyphosphate as a novel flame retardant system for cotton fabrics. Chem. Eng. J. **451**, 138566 (2023). 10.1016/j.cej.2022.138566

