# Supplementary material for: Layered Double Hydroxide Nanosheets Incorporated Hierarchical Hydrogen Bonding Polymer Networks for Transparent and Fire-Proof Ceramizable Coatings
Source: Nanomicro Lett. 2025 Jan 27;17:116. doi: 10.1007/s40820-025-01646-y (PMC11769928; doi:10.1007/s40820-025-01646-y)
Supplement: Supplementary file 22 — Supplementary file22 (DOCX 28620 KB) [file 40820_2025_1646_MOESM22_ESM.docx]

Supporting Information for

**Layered Double Hydroxide Nanosheets Incorporated Hierarchical Hydrogen Bonding Polymer Networks for Transparent and Fire-Proof Ceramizable Coatings**

Bifan Guo^1,^ ^ǂ^, Yimin He^1, ǂ^, Yongming Chen^1^, Tianci Yang^1^, Chaohua Peng^1^, Weiang Luo^1,2^, Birong Zeng^1,2^, Yiting Xu^1,2^, Lizong Dai^1, 2,^ *

^1^ Fujian Provincial Key Laboratory of Fire Retardant Materials, College of Materials, Xiamen University, Xiamen 361000, P. R. China

^2^ Xiamen Key Laboratory of Fire Retardant Materials, Xiamen University, Xiamen 361000, P. R. China

ǂ Bifan Guo and Yimin He contributed equally to this work.

*Corresponding author. E-mail: [lzdai@xmu.edu.cn](mailto:lzdai@xmu.edu.cn) (Lizong Dai)

**S1 Experimental Section**

**S1.1 Characterizations**

The morphology and microstructure of LDHs and various samples were analyzed using transmission electron microscope (TEM, Talos F200s, Netherlands) and scanning electron microscopy (SEM) equipped with an energy-dispersive spectrometer (EDS) on a Sigma-500, ZEISS instrument. The ^1^H cross-polarization magic angle spinning (CP/MAS) nuclear magnetic resonance (NMR) measurements were conducted on a 500 MHz NMR spectrometer from Bruker Corp., Germany. The chemical compositions and structure of the materials were analyzed using a Nicolet 7000 Fourier-transform infrared (FT-IR) spectrometer, Raman spectra (Xplora, HORIBA, France), and an X-ray photoelectron spectrometer (XPS, Thermo Fisher Escalab Xi^+^, America), respectively. UV-vis transmission spectra were obtained using a Perkin-Elmer Lambda 35 UV-vis spectrometer (Perkin-Elmer, USA). Uniaxial stress-strain tests were performed using a dynamic mechanical thermal analyzer (DMA-Q800) from TA Instruments, USA. The tension rate was 2.0 mm/min, with at least five specimens were tested per batch to obtain an average value. Shear adhesion strength was evaluated using a universal testing machine (AGS-X 5KN) according to the ASTM F2255 standard. X-ray diffraction (XRD) analysis was performed using a D8-A25 X-ray diffractometer with a 2θ range of 5° to 80° (Bruker, Germany). The water contact angle of the samples was measured at room temperature using a DSA30 CA analyzer from Kruss, Germany. Thermographic images were captured using an infrared thermal camera (FLIR E60) with a thermal sensitivity of ≤2% (Fluke, America).

Temperature-dependent FTIR spectra of PSH/BM/LDHs film from 25 to 55 °C were used for performing 2D correlation analysis. The 2D correlation analysis was carried out using the software 2D Shige ver. 1.3 (©Shigeaki Morita, Kwansei-Gakuin University, Japan, 2004-2005), and further plotted into contour maps using the Origin program. In the contour maps, warm colors (red) are defined as positive intensities, while cold colors (blue) represent negative intensities.

The thermal stability of the samples was tested using TGA-8000 thermogravimetry analyzer (PerkinElmer, USA). Samples (5-10 mg) were heated from 30 °C to 800 °C at a heating rate of 10 °C/min under an air/N₂ atmosphere with a gas flow of 50 mL/min. The pyrolysis products were monitored by TGA coupled with FTIR (TGA-FTIR) and TGA coupled with MS (TGA-MS). For TGA-FTIR testing, a TGA-8000 (PerkinElmer, USA) was combined with an FTIR (PerkinElmer, USA) infrared spectrometer. Samples (5-10 mg) were heated from 30 °C to 800 °C at a heating rate of 10 °C/min under an air/N₂ atmosphere with a gas flow of 50 mL/min. For TGA-MS testing, the gaseous products were analyzed by gas chromatography-mass spectrometry (GC-MS, PerkinElmer, USA) under the same conditions as TGA-FTIR (air).

The limited oxygen index (LOI) was measured using a JF-3 oxygen index instrument (Jiangning, China) with sample dimensions of 150×10×10 mm^3^, following the ASTMD-2863 standard. The average values from three samples were recorded. The UL-94 vertical burning test was conducted using a CZF-3 instrument (Nanjing Jiangning Analytical Instruments Co., Ltd., China) according to ASTM D 3801, and the average values of three samples were recorded. A cone calorimeter (Fire Testing Technology, UK) was used to evaluate the combustion performance of the FPUF filled with potassium salt according to ISO 5660-1, with wood specimens (100×100×10 mm^3^) tested at a heat flux of 50 kW/m^2^. The smoke toxicity gas concentration was measured using an FTT0095 smoke toxicity tester (Fire Testing Technology, UK) according to ISO 5659-2.

**S2 Supplementary Figures and Tables**

**
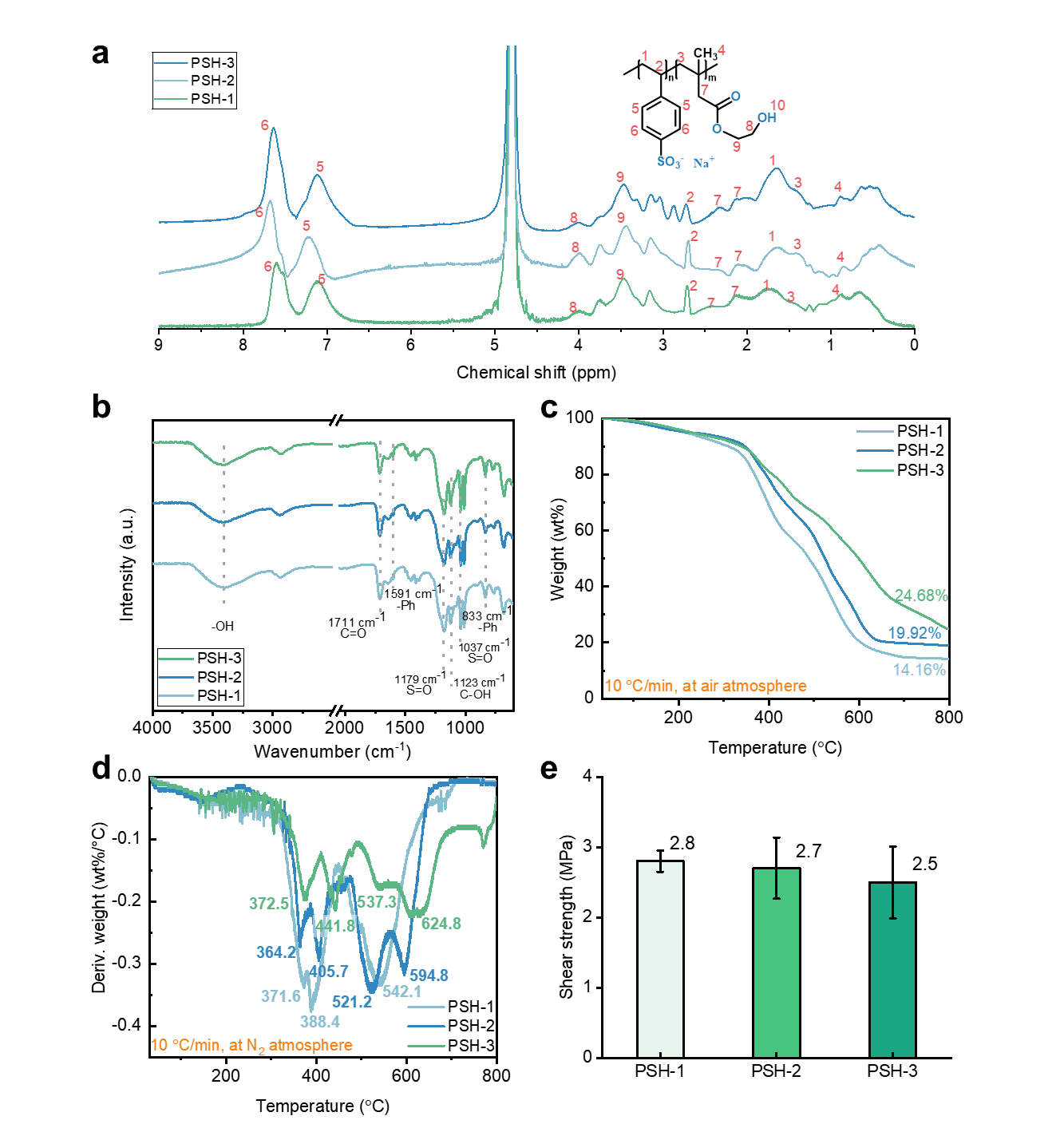
**

**Fig. S1** (**a**) The ^1^H NMR spectra, (**b**) FTIR spectra and assignments, (**c-d**) TGA and DTG curves at air atmosphere, and (e) shear strength for wood of poly(SSS-HEMA) with various SSS/HEMA ratios

To confirm the chemical structure and composition of the three copolymers, infrared spectra were conducted (Fig. S1a). Notably, a broad absorption peak at ~3407 cm^-1^ and a sharp absorption peak at 1711 cm^-1^ are attributed to the hydroxyl groups (C-OH) and carbonyl (C=O) of HEMA unit, respectively. Additionally, characteristic peaks at 1179 cm^-1^ (S=O symmetric vibrational stretching), 1037 cm^-1^ (S=O asymmetric vibrational stretching), and 1591 cm^-1^ (-Ph) for SSS unit.

**
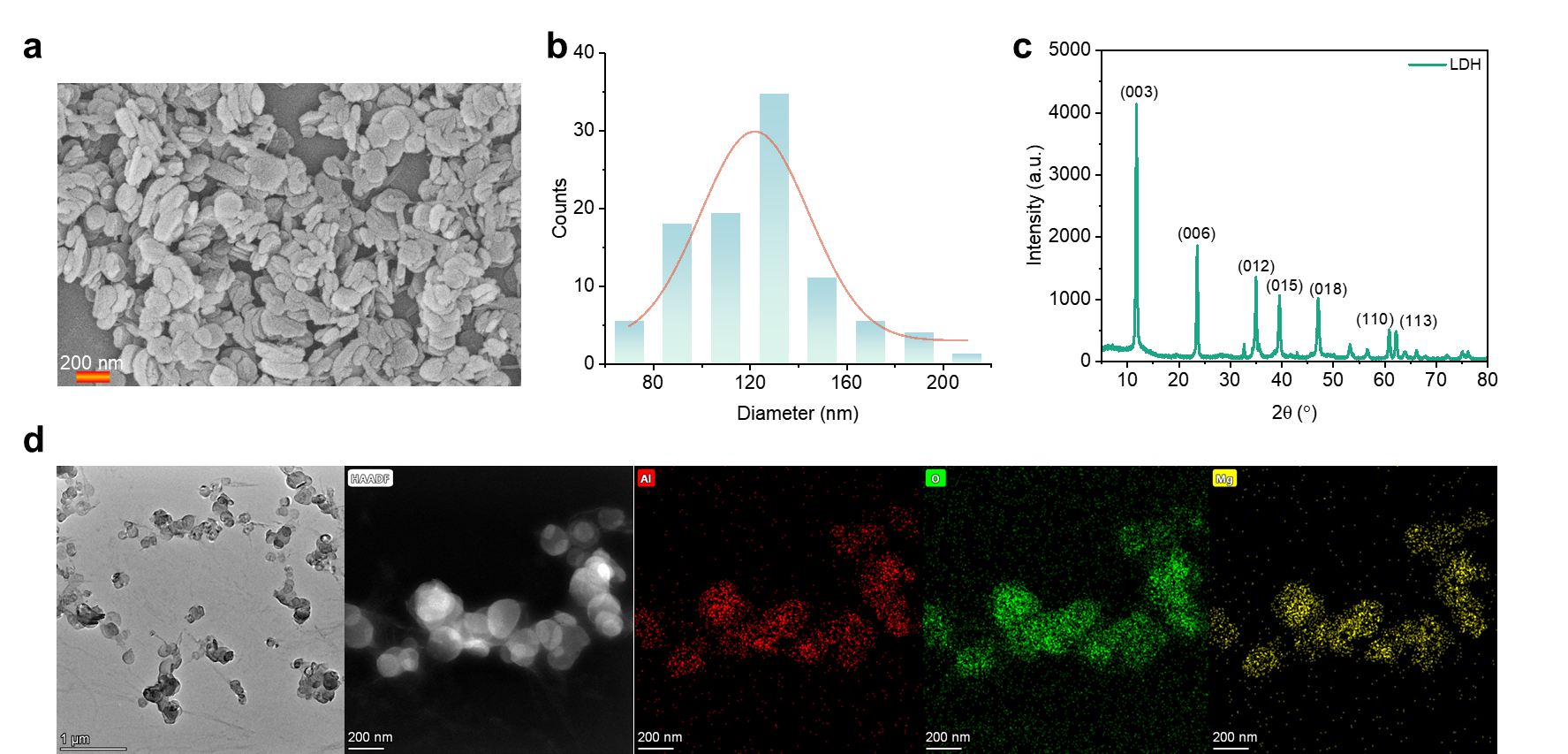
**

**Fig. S2** (**a**) SEM image, (**b**) corresponding size distribution, (**c**) XRD patterns of LDHs, and (**d**) TEM images as well as corresponding to mapping images

**
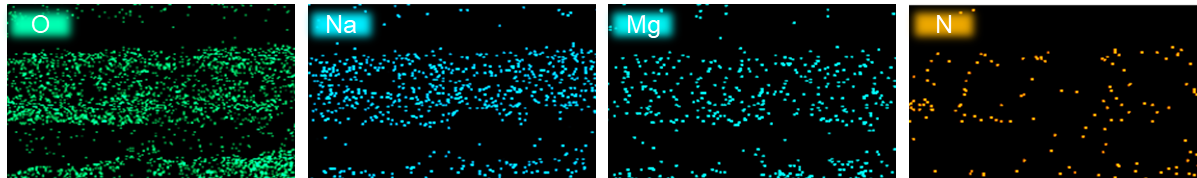
**

**Fig. S3** EDS elemental mapping images for O, Na, Mg, and N from the cross-section of the PSH/BM/LDH-coated wood

**
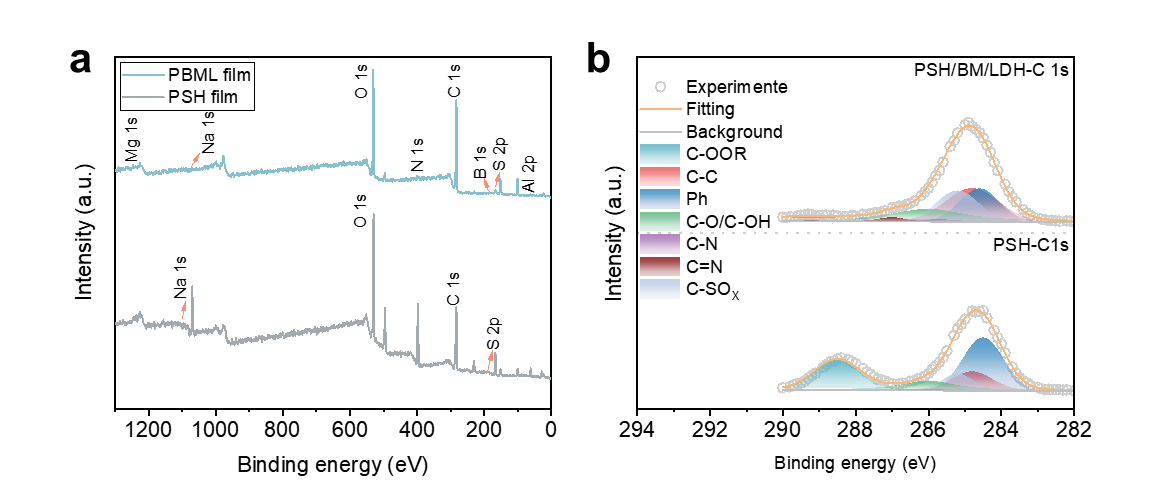
**

**Fig. S4** (**a**) XPS results and (**b**) XPS C 1s spectra of PSH and PBML film

**
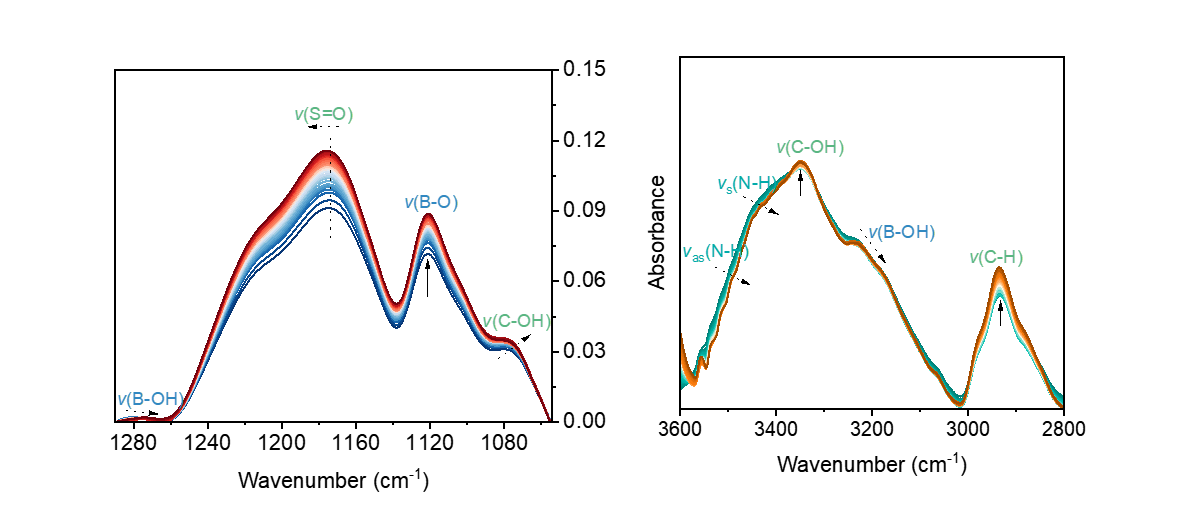
**

**Fig. S5** Temperature-variable FTIR spectra of PSH/BM/LDHs films upon heating from 25 to 55 °C in the regions of (interval: 1 °C)

**
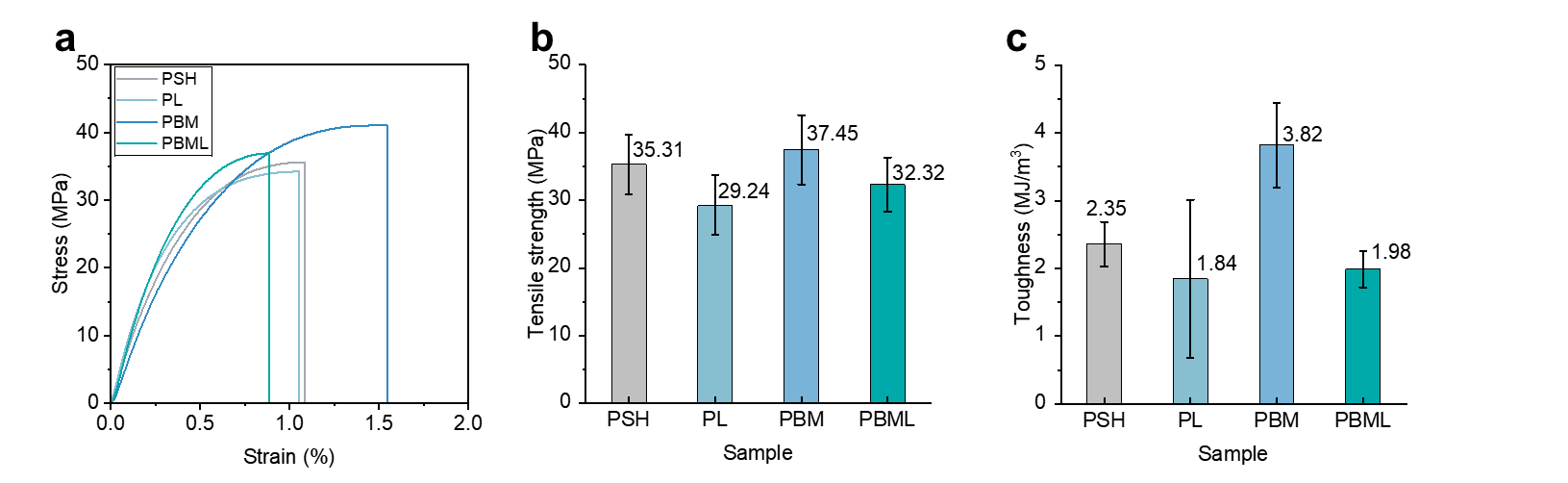
**

**Fig. S6** (**a**) The stress-strain curves, (**b**) tensile strength, (**c**) and toughness values of various paper samples

**
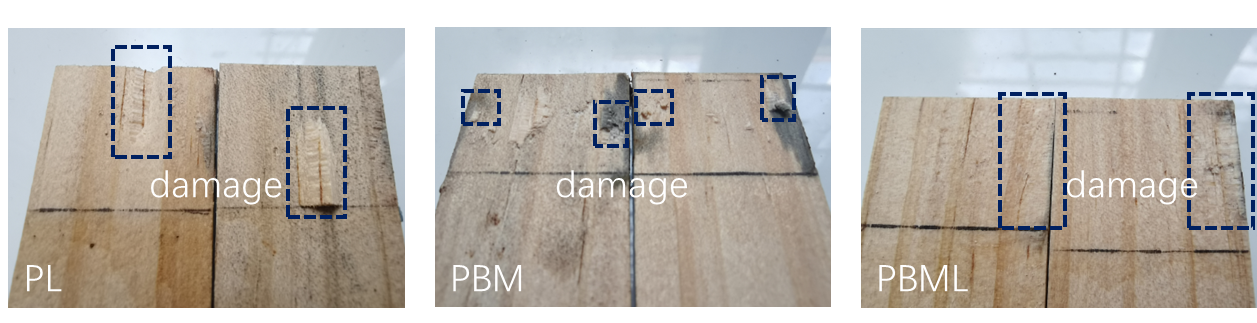
**

**Fig. S7** Digital image of PL, PBM, and PBML coatings against wood after shear tests

**
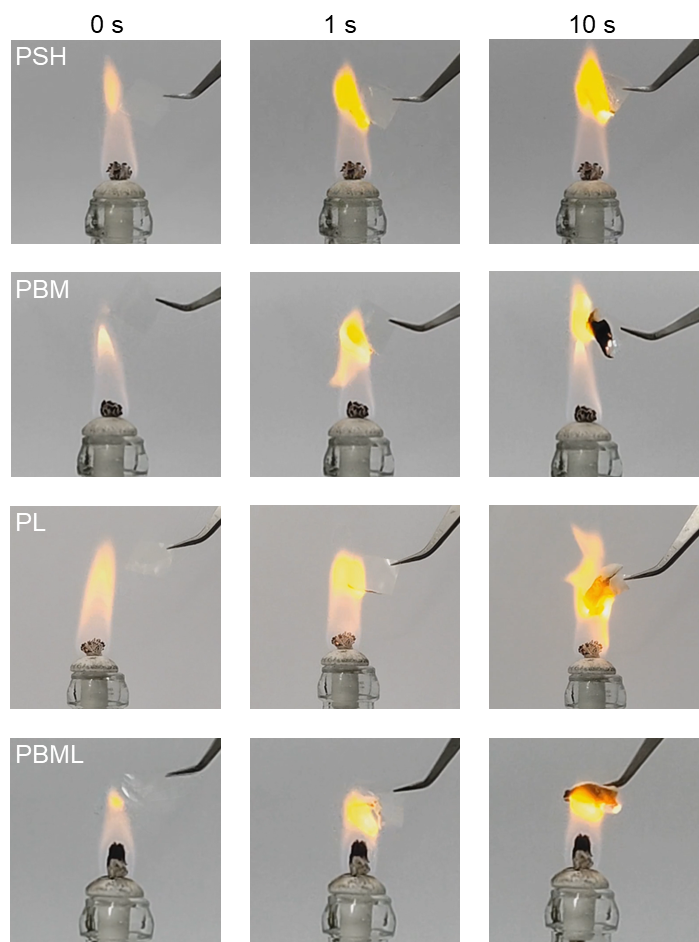
**

**Fig. S8** Combustion process of PSH, PBM, PL, and PBML papers

**
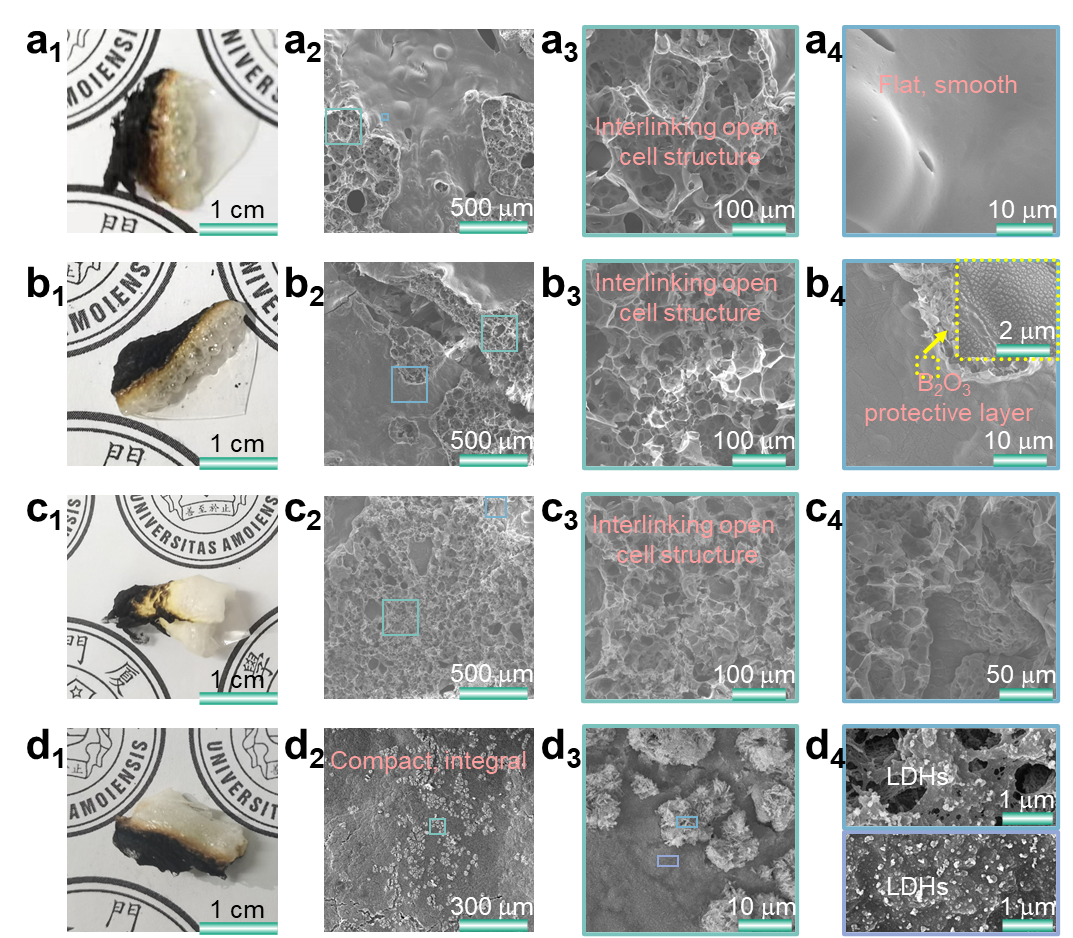
**

**Fig. S9** (**a_1_-d_4_**) Optical image and SEM of PSH, PBM, PL, and PBML papers after combustion


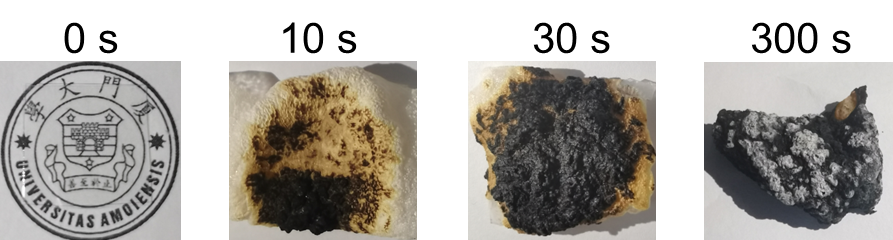


**Fig. S10** Digital images for the top surface of PBML paper after being exposed to the alcohol lamp flame (~500 °C) for different time


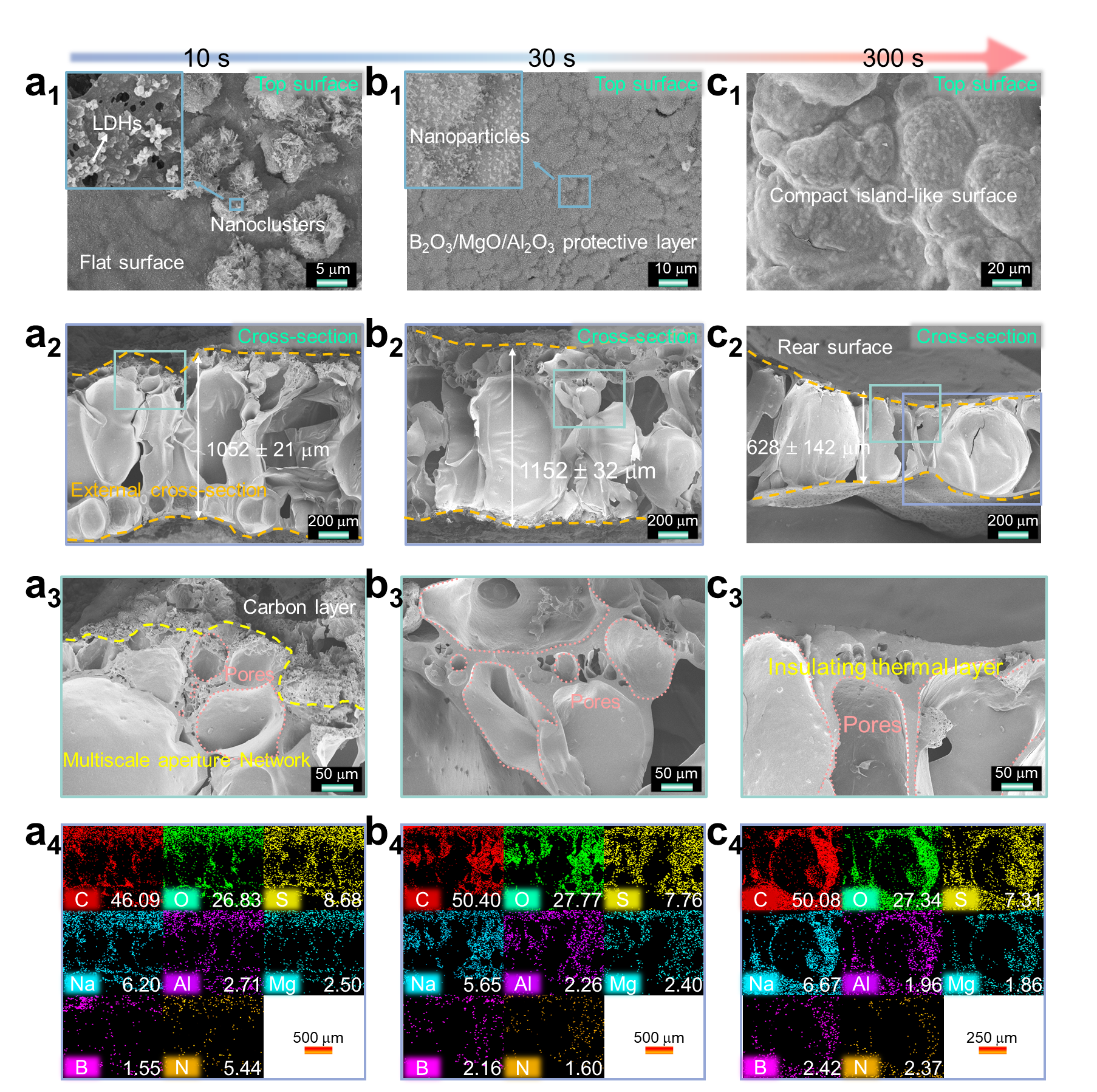


**Fig. S11** SEM images (EDS mapping) of the PBML paper: (**a_1_-c_1_**) the top surface and (**a_2_-c_3_**) the cross-section after being exposed to the alcohol lamp flame (~500 °C) for different time (e.g., 10 s, 30 s, and 300 s) as well as (**a_4_-c_4_**) corresponding EDS mapping

**
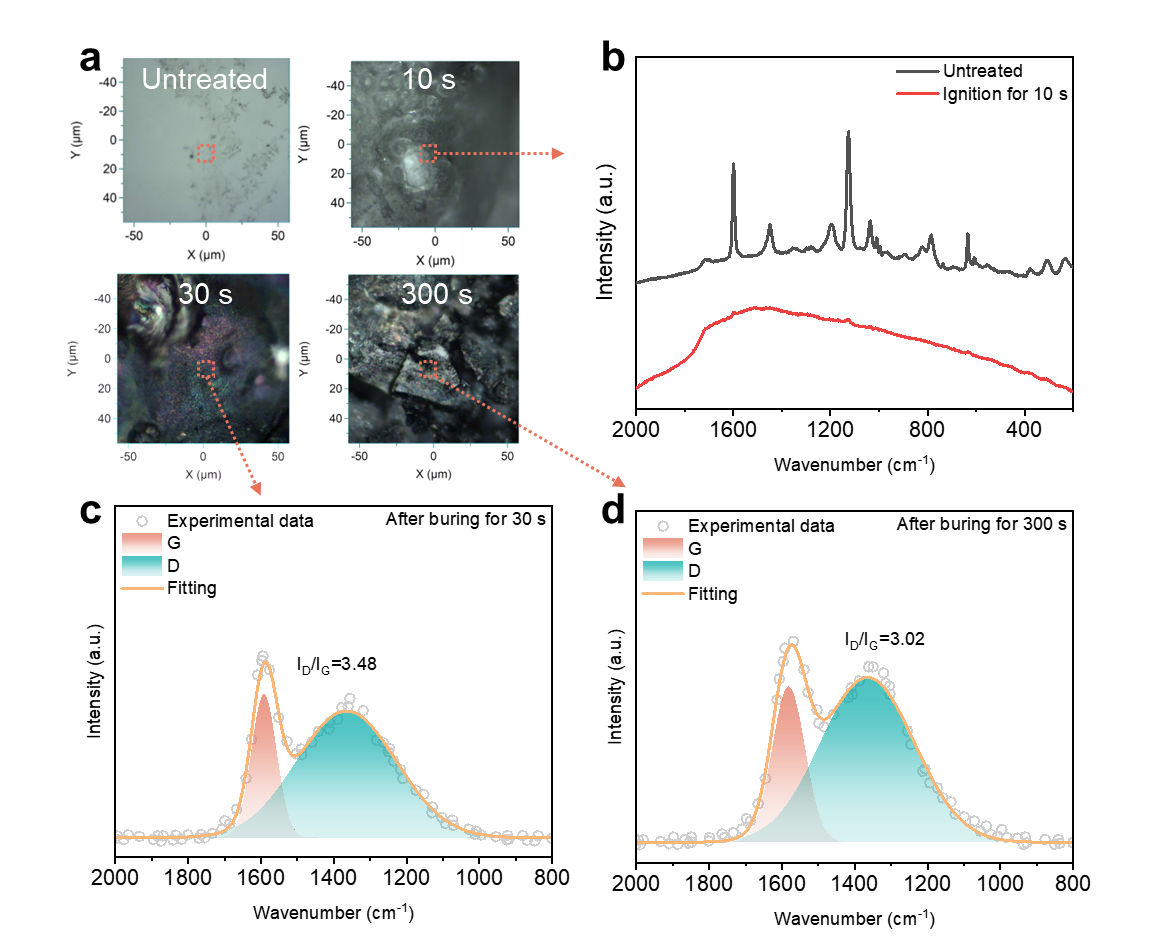
**

**Fig. S12** (**b-d**) Raman spectra of the top surface materials after fire treatment with different time, and (**a**) corresponding to microscope images of the test point

**
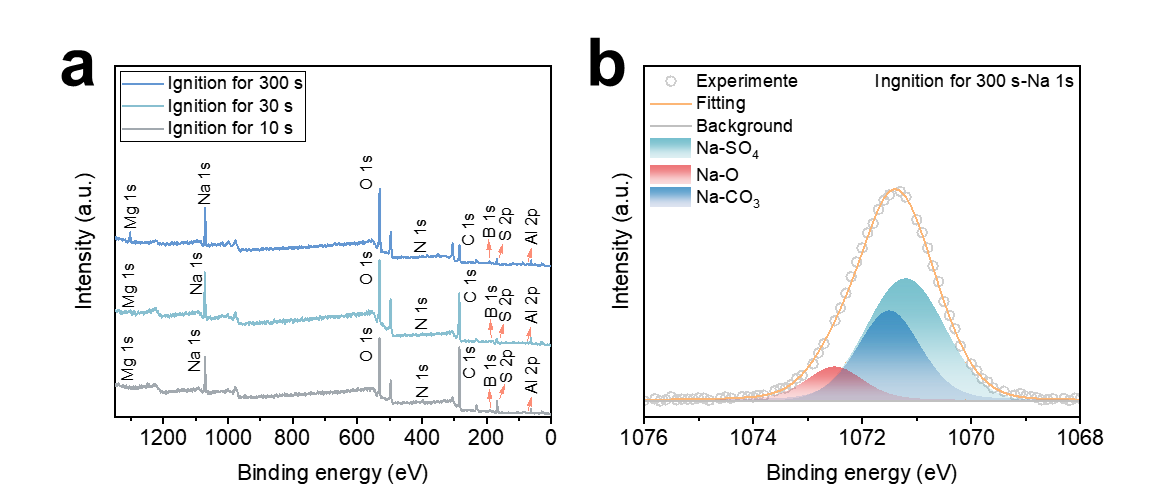
**

**Fig. S13** (**a**) XPS results and (**b**) XPS Na1s of PBML film after burning for different time

**
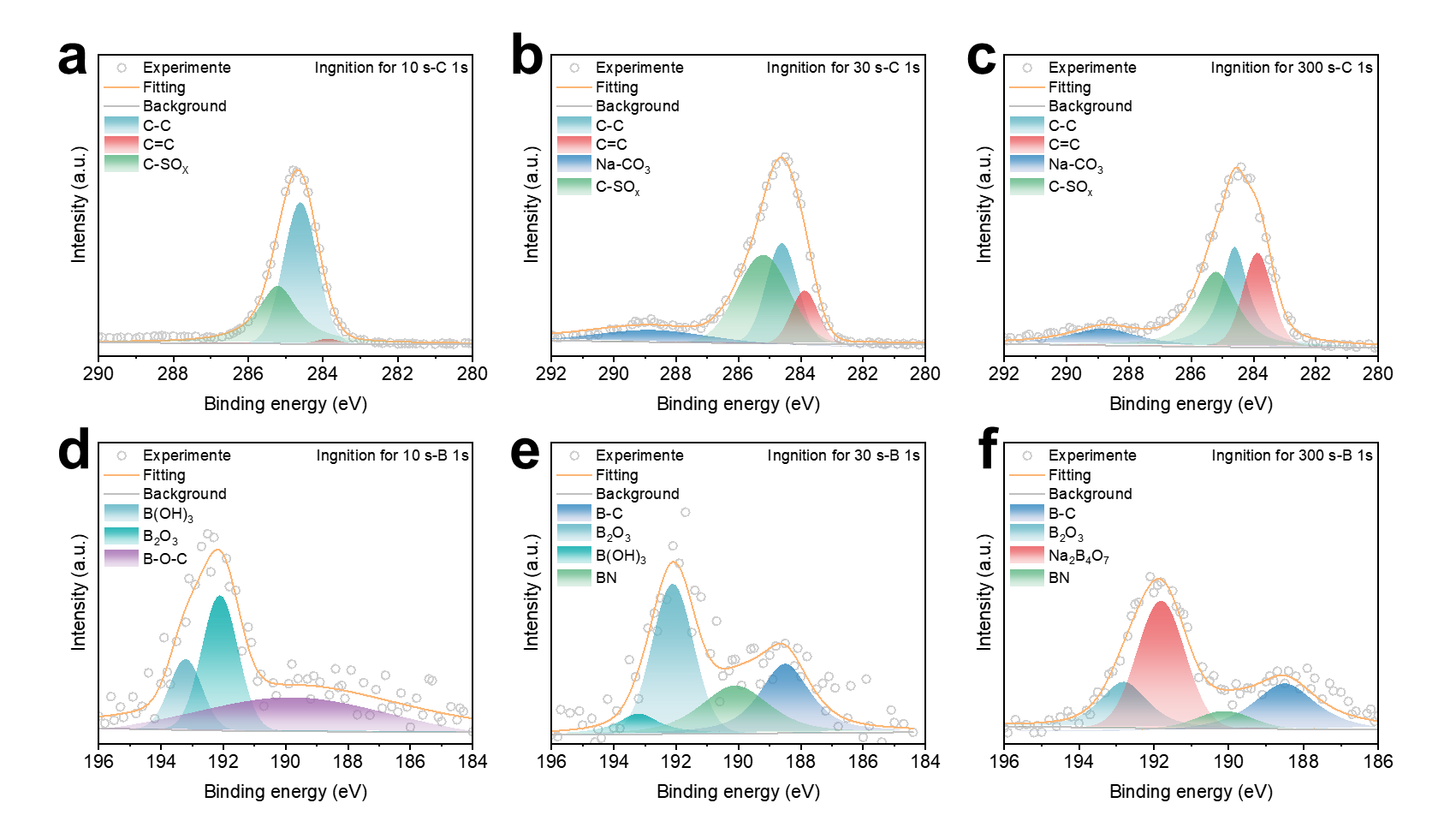
**

**Fig. S14** (**a-f**) XPS C 1s, and B 1s spectra of PBML film after being flame attacked with different time

**
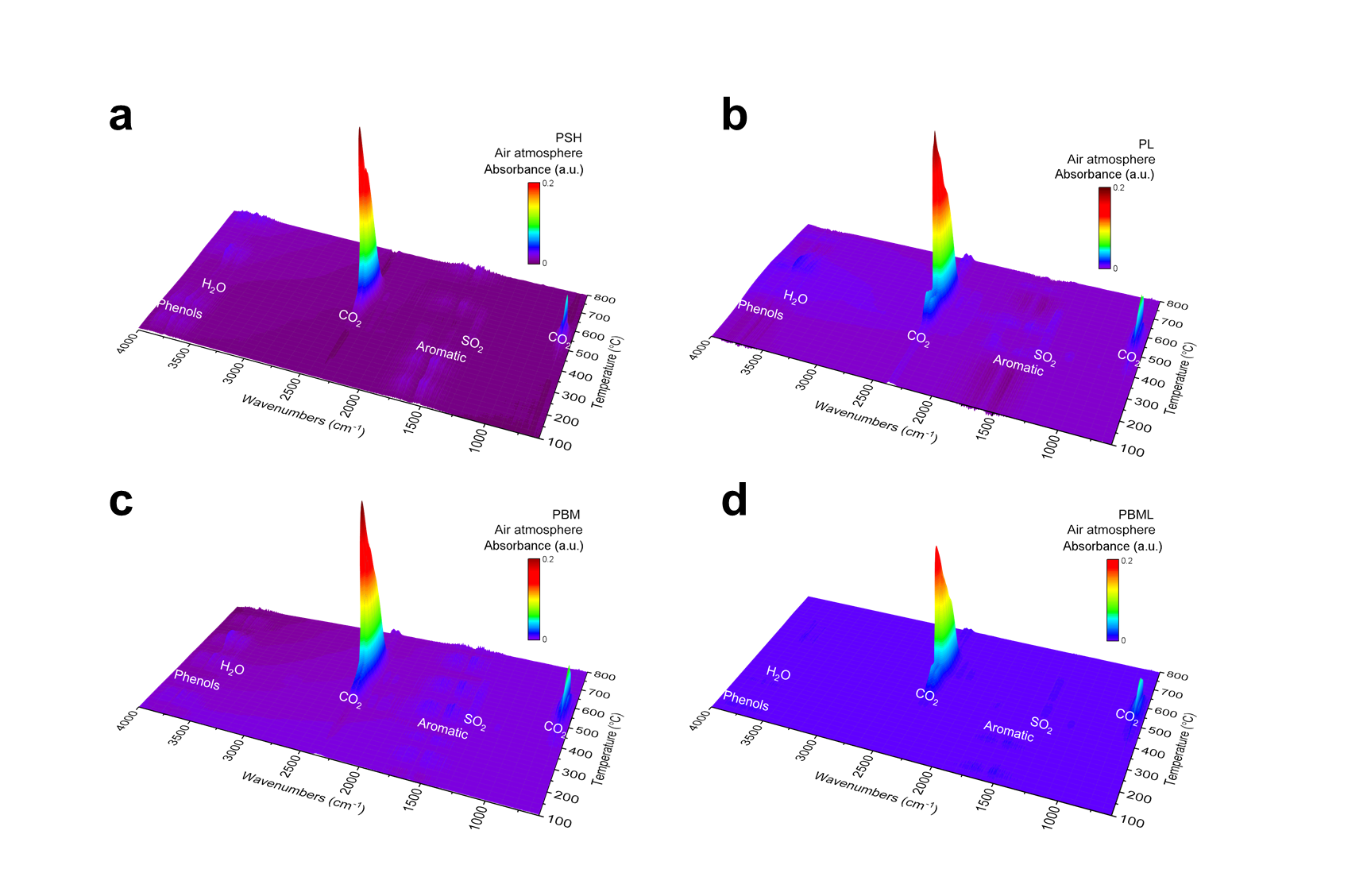
**

**Fig. S15** (**a-d**) The three-dimensional spectra of pyrolysis products of PSH, PL, PBM, and PBML papers under air atmosphere

**
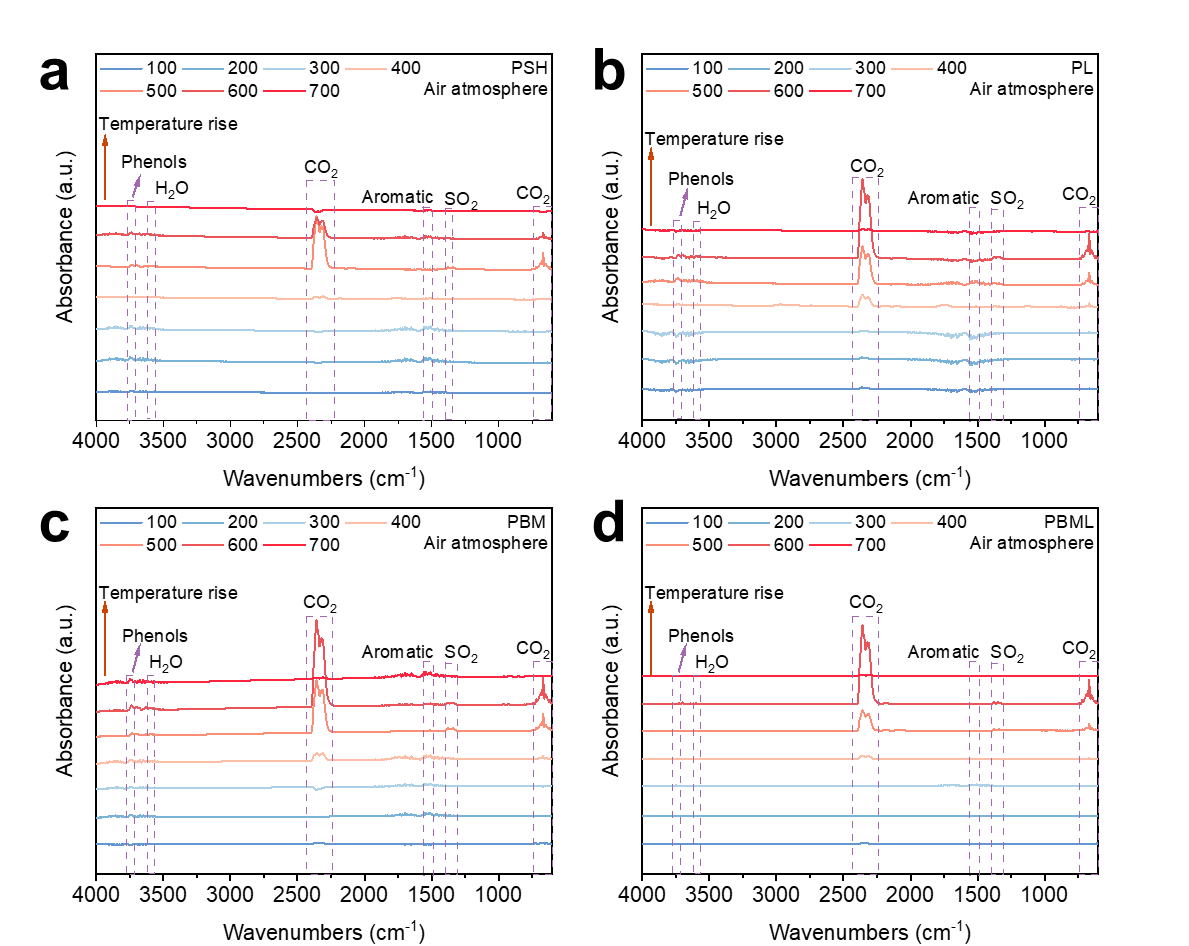
**

**Fig. S16** (**a-d**) The infrared spectra of PSH, PL, PBM, and PBML under air atmosphere at various temperatures

**
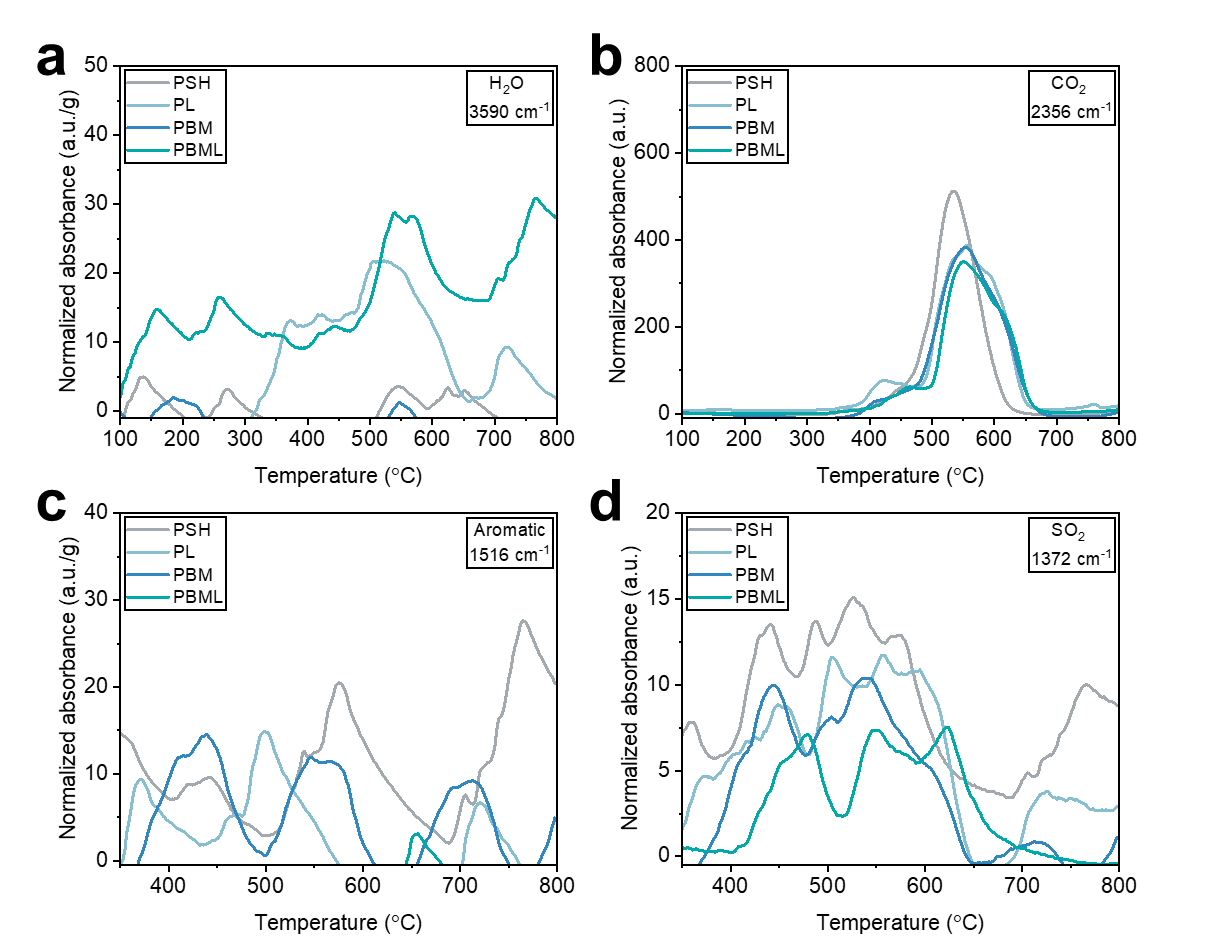
**

**Fig. S17** Normalized absorbance curves of (**a**) SO_2_, (**b**) aromatic, (**c**) CO_2_, and (**d**) H_2_O during pyrolysis of PBML composites obtain from TGA coupled with FTIR during 350-800 °C under air atmosphere. The normalized absorbance (a.u./g) of each sample is calculated by dividing the measured absorbance by its own mass

**
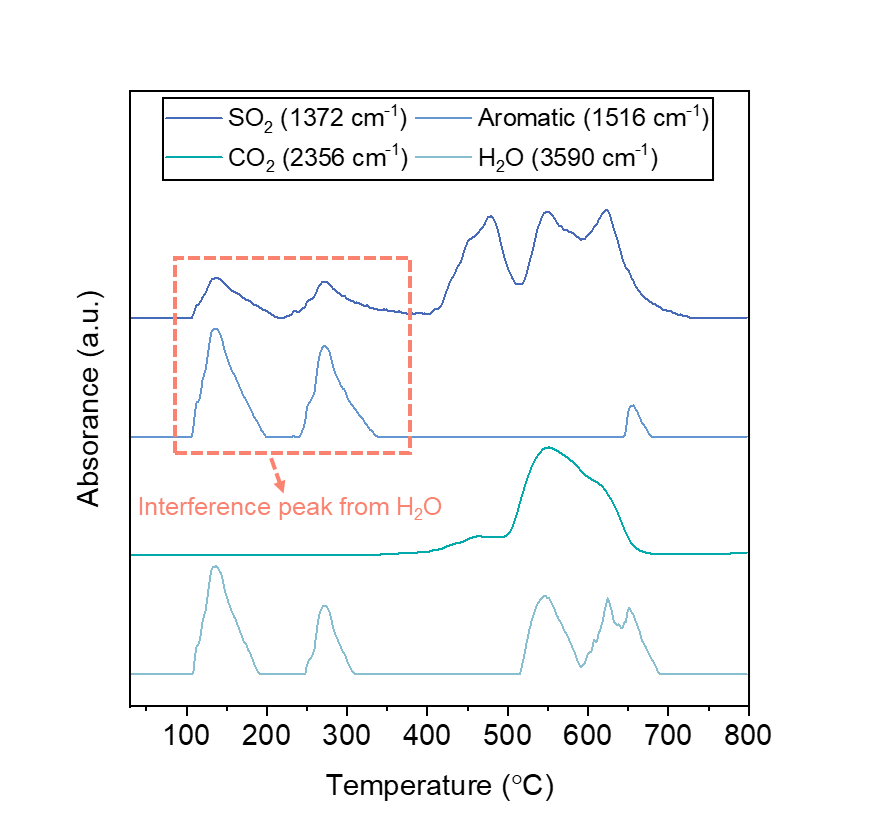
**

**Fig. S18** The curves of SO_2_, aromatic, CO_2_, and H_2_O during pyrolysis of PBML composites obtain from TGA coupled with FTIR during 30-800 °C under air atmosphere


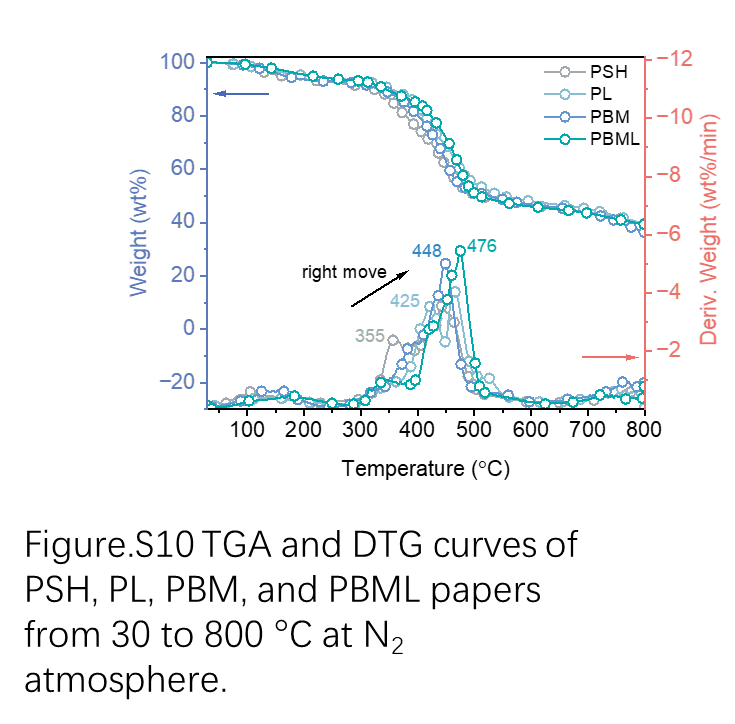


**Fig. S19** TGA and DTG curves of PSH, PL, PBM, and PBML papers from 30 to 800 °C at N_2_ atmosphere

**
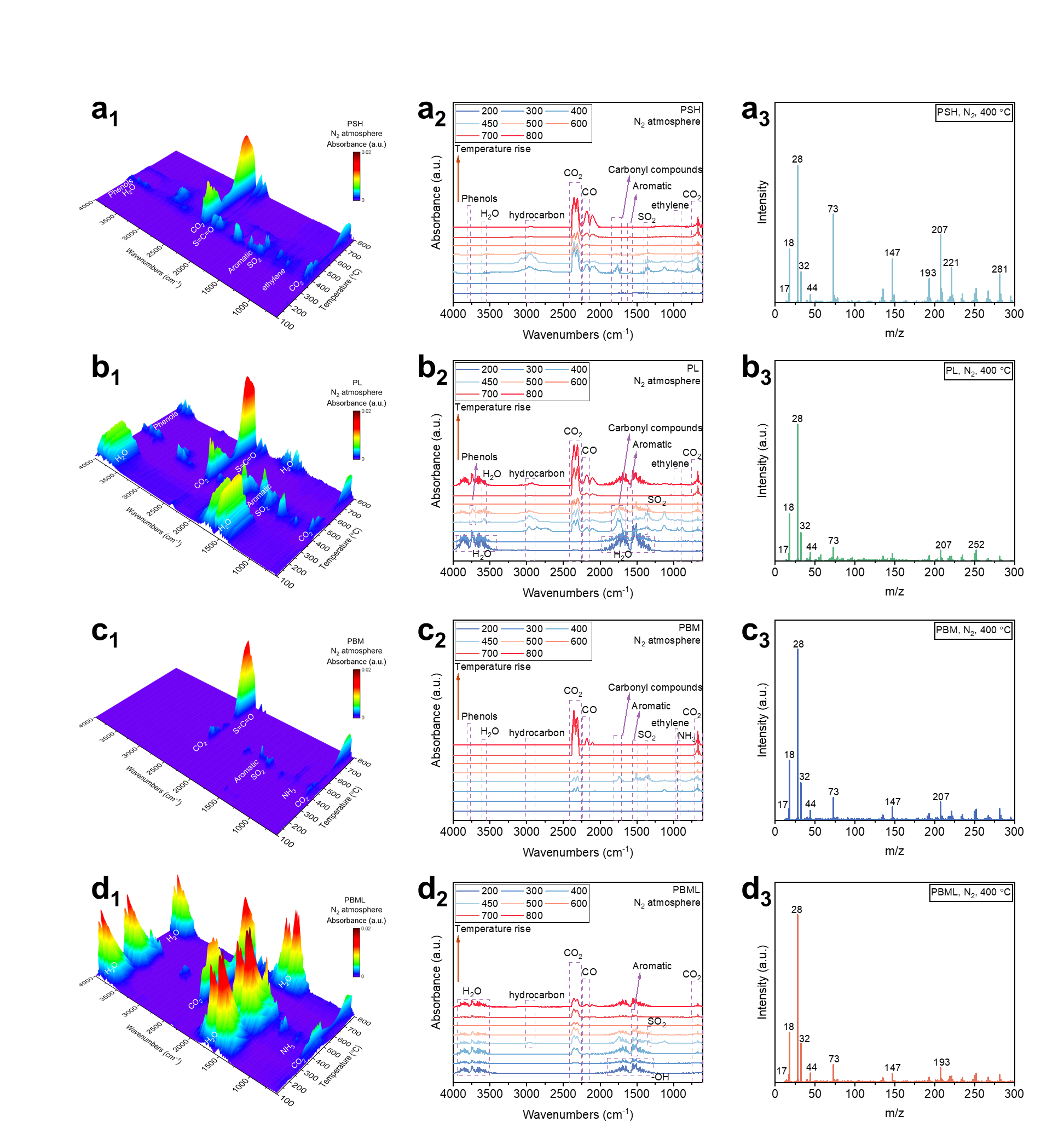
**

**Fig. S20** (**a_1_-d_1_**) The three-dimensional spectra of pyrolysis products of PSH, PL, PBM, and PBML papers under air atmosphere. (**a_2_-d_2_**) The infrared spectra of PSH, PL, PBM, and PBML under N_2_ atmosphere at various temperatures. (**a_3_-d_3_**) The pyrolysis products of PSH, PL, PBM, and PBML obtained from TGA coupled with GC-MS at 400 °C under N_2_ atmosphere


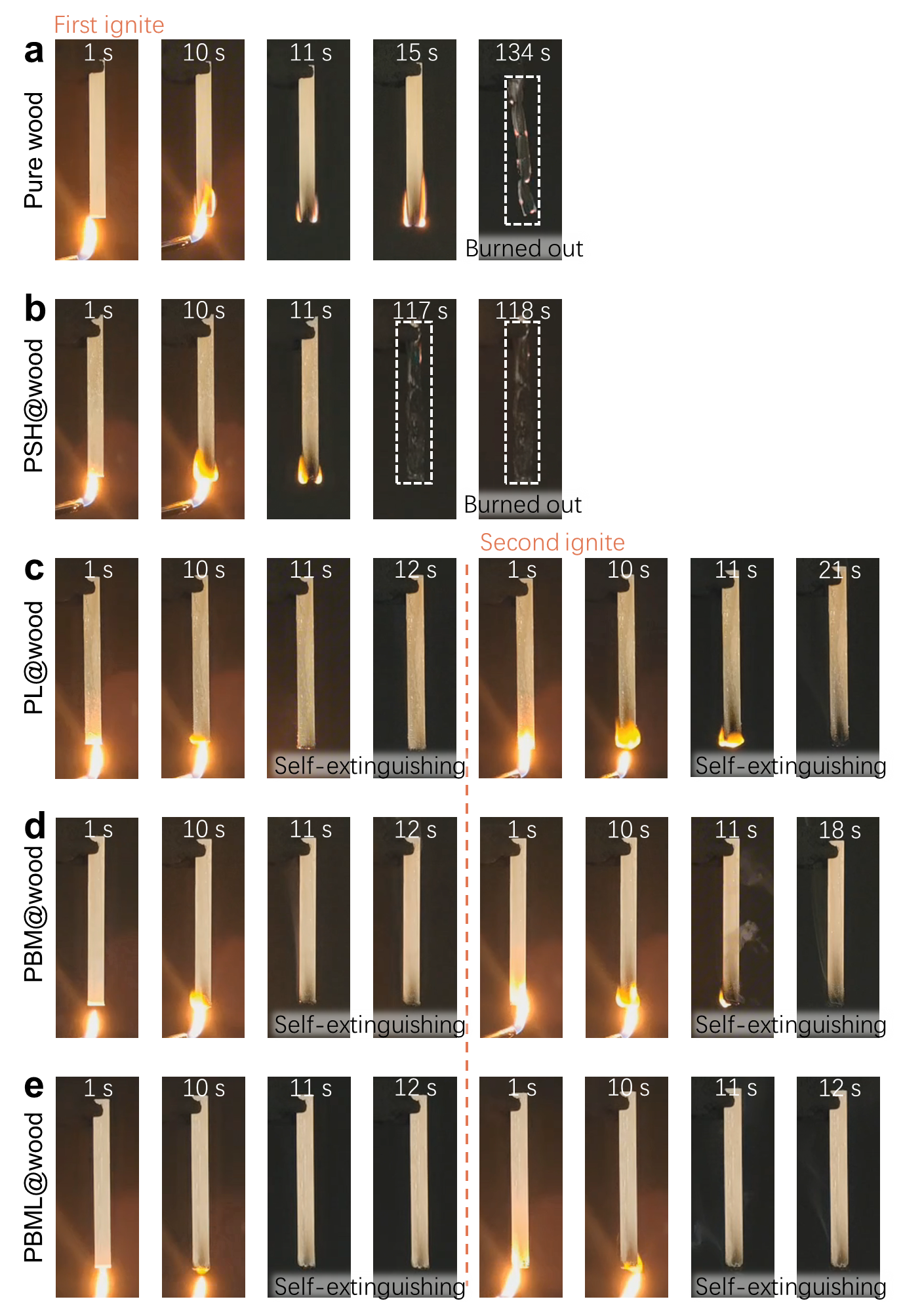


**Fig. S21** (**a-e**) Optical photos showing the burning process of wood and coated woods as a function of time during UL-94 tests

**
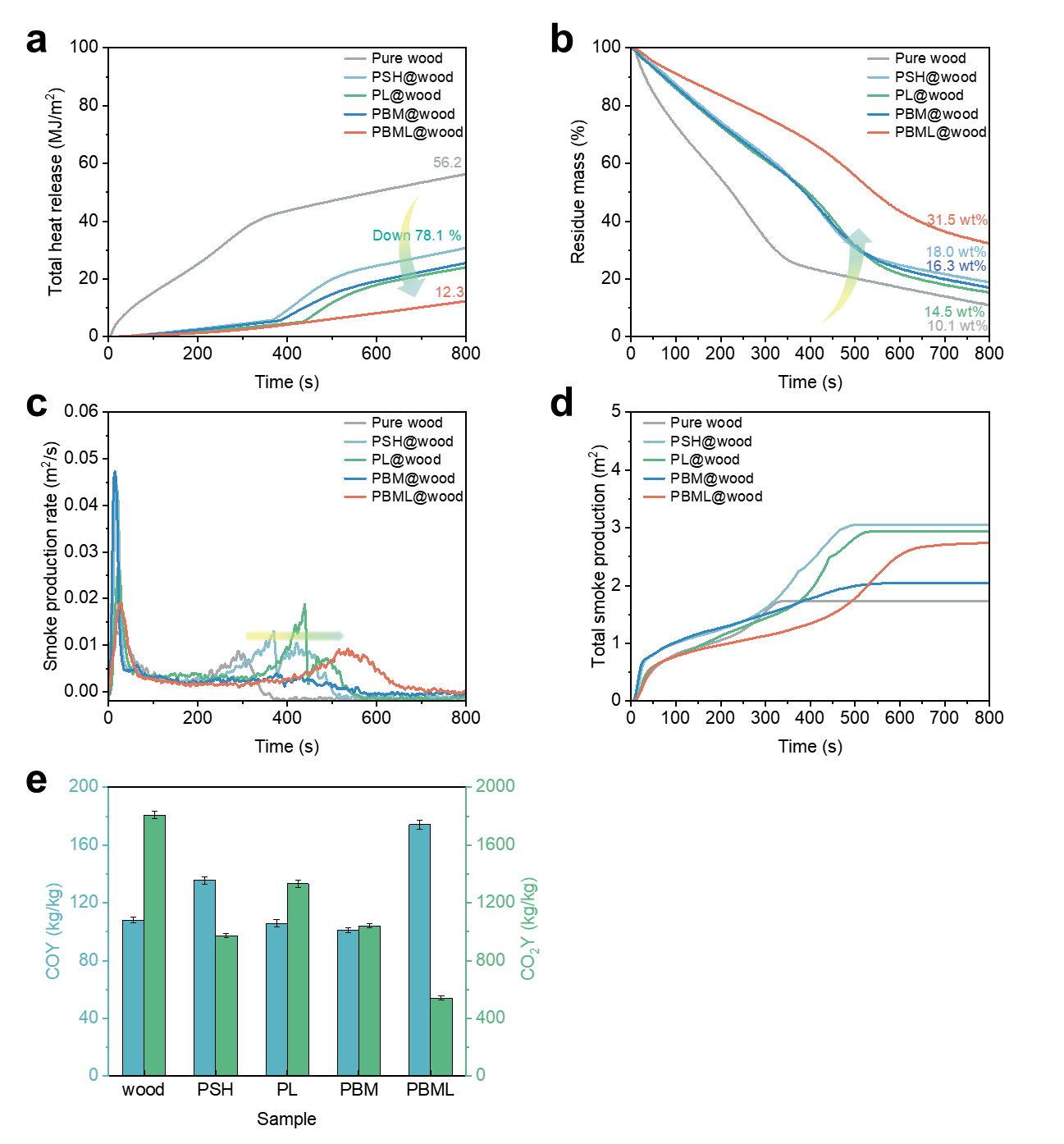
**

**Fig. S22** (**a-e**) THR, residue mass, SPR, TSP, and COY and CO_2_Y of various samples

**
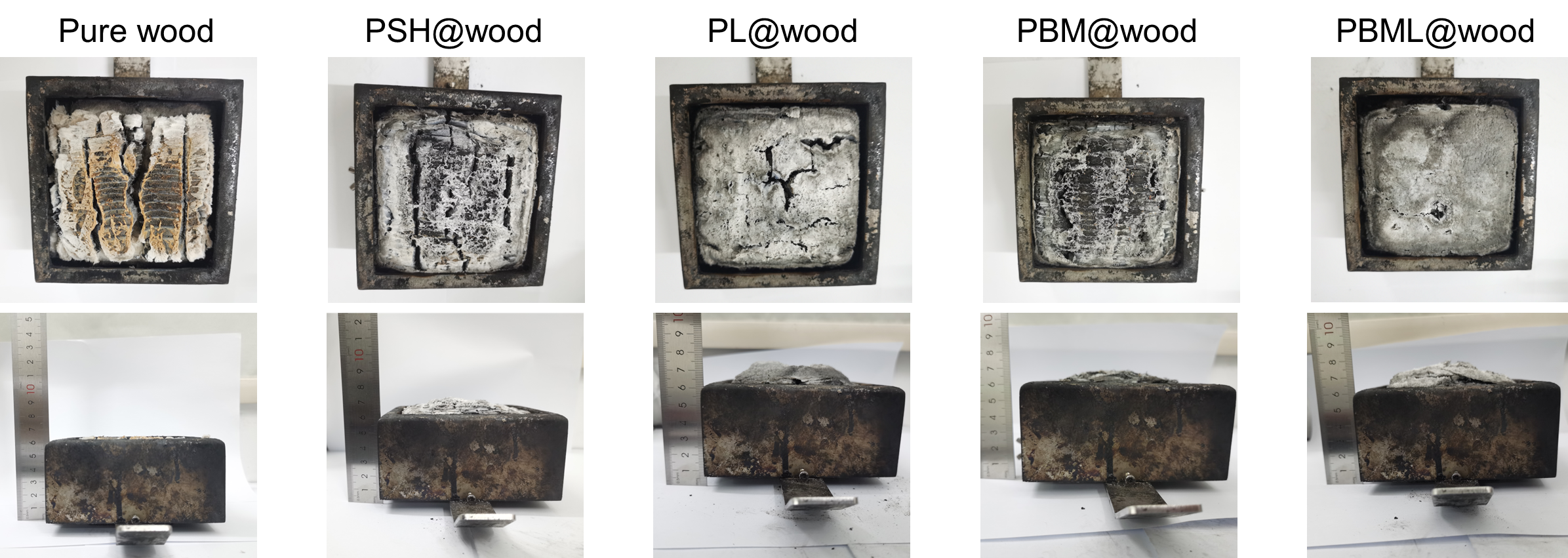
**

**Fig. S23** (**a-e**) Digital images of residue chars for (**a**) pure wood, (**b**) PSH@wood, (**c**) PL@wood, (**d**) PBM@wood, and (**e**) PBML@wood after cone testing

**
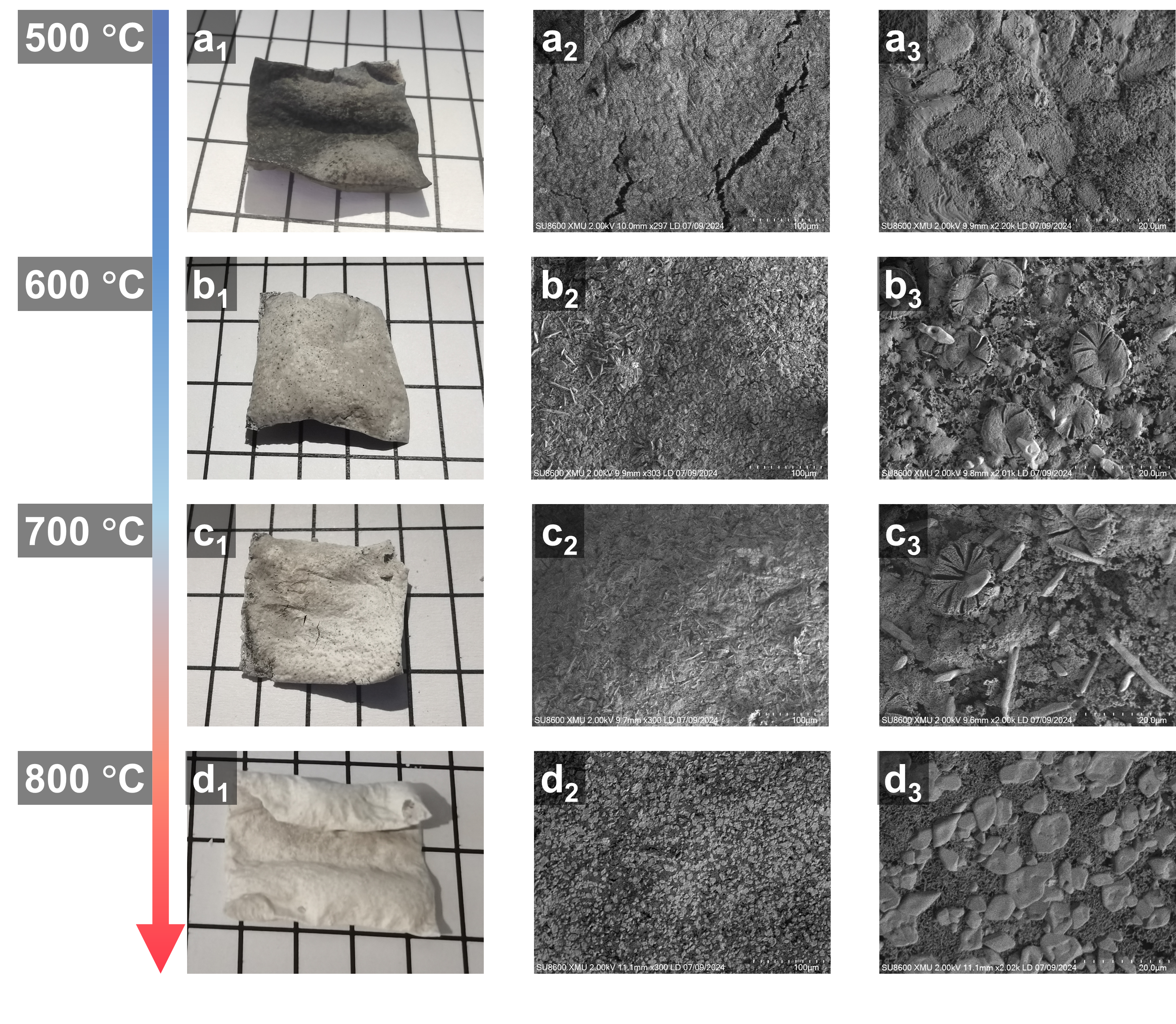
**

**Fig. S24** (**a_1_-d_1_**) Digital photos and (**a_2_-d_3_**) corresponding SEM images of the coating top surface with increasing heat-temperature, showing the evolution of an integral compact char

**
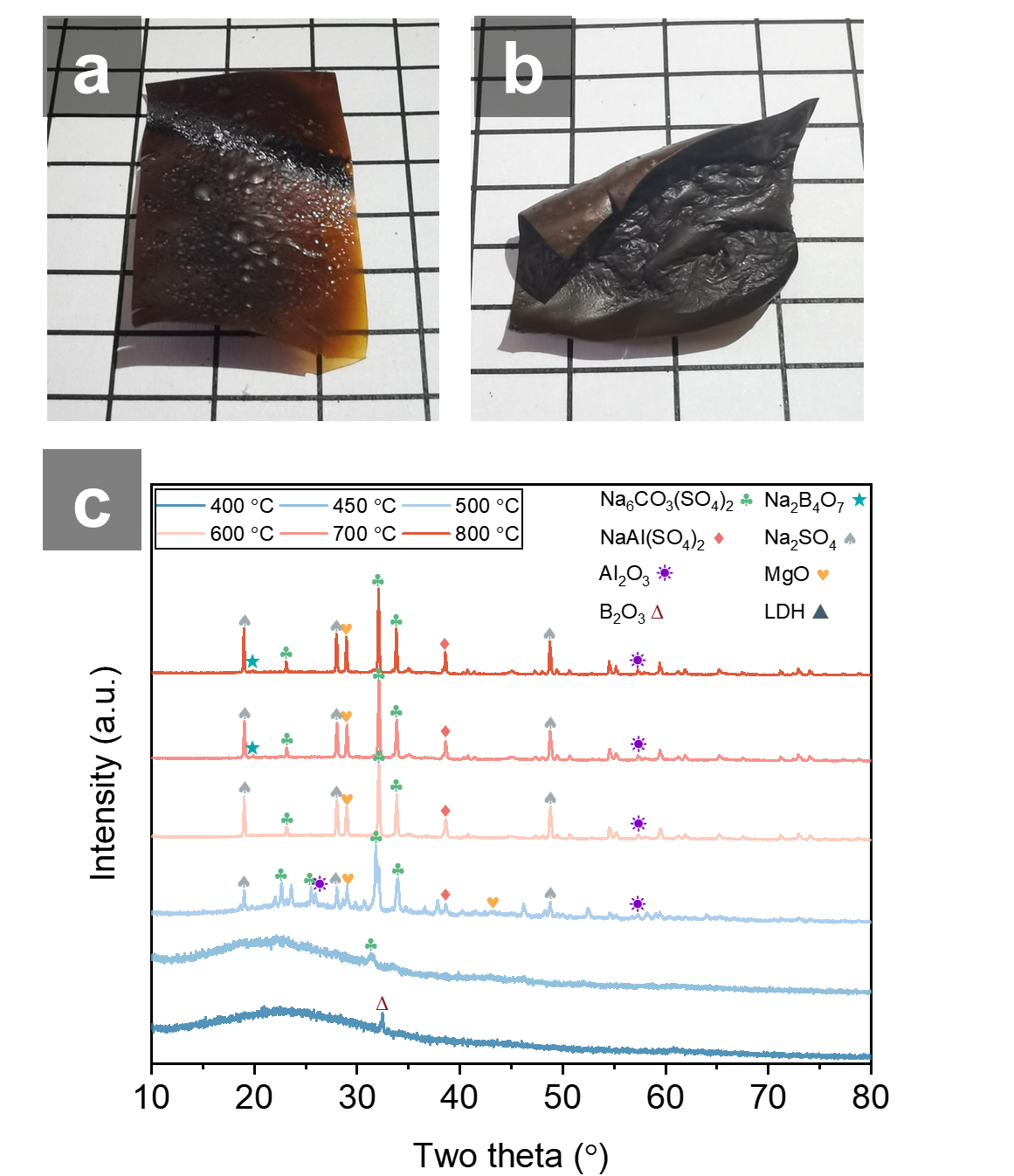
**

**Fig. S25** (**a-b**) Digital photos PBML films 400 °C and 450 °C after heat treatment, respectively. (**c**) The XRD patterns of the coating top surface with increasing heat-temperature

Note: In general, the variation trend of the samples treated with temperature is consistent with that of the samples exposed to flame. As the temperature increases, the PBML film gradually carbonizes to form char, which then slowly transforms into a white, expanded, ceramic-like appearance (Fig. S24a_1_-d_1_ and Fig.S25a-b). In addition, SEM images show the formation of nanoclusters and their gradual integration into the carbon layer, together forming a stronger fire-proof protective layer (Fig. S24a_2_-d_3_). Similarly, XRD patterns further confirm this result, indicating that the amorphous carbon layer was gradually covered by inorganic salts or inorganic oxides (Fig. S25c).

**
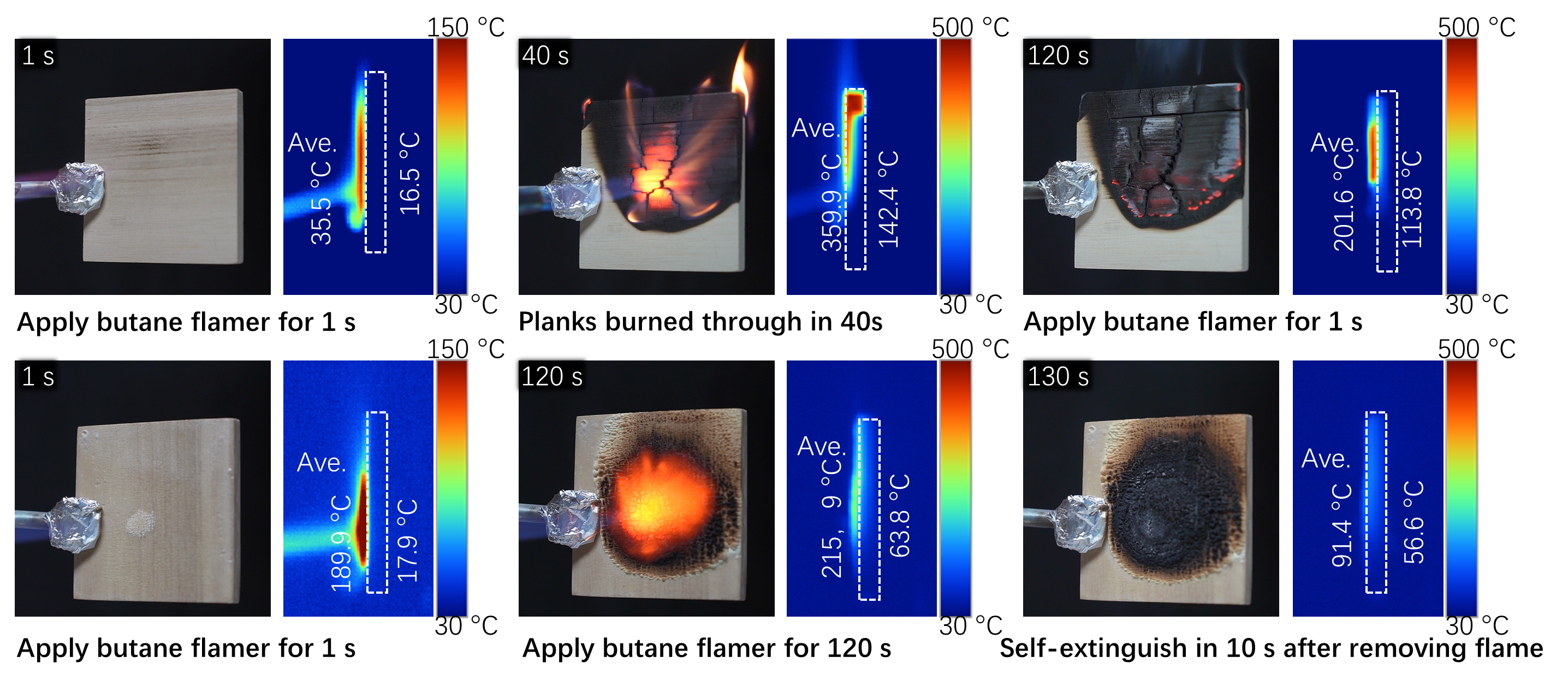
**

**Fig. S26** (**a**) wood and (**b**) PBML@wood under the butane flame for 120 s, along with their side temperature variation with time determined by the IR camera

**
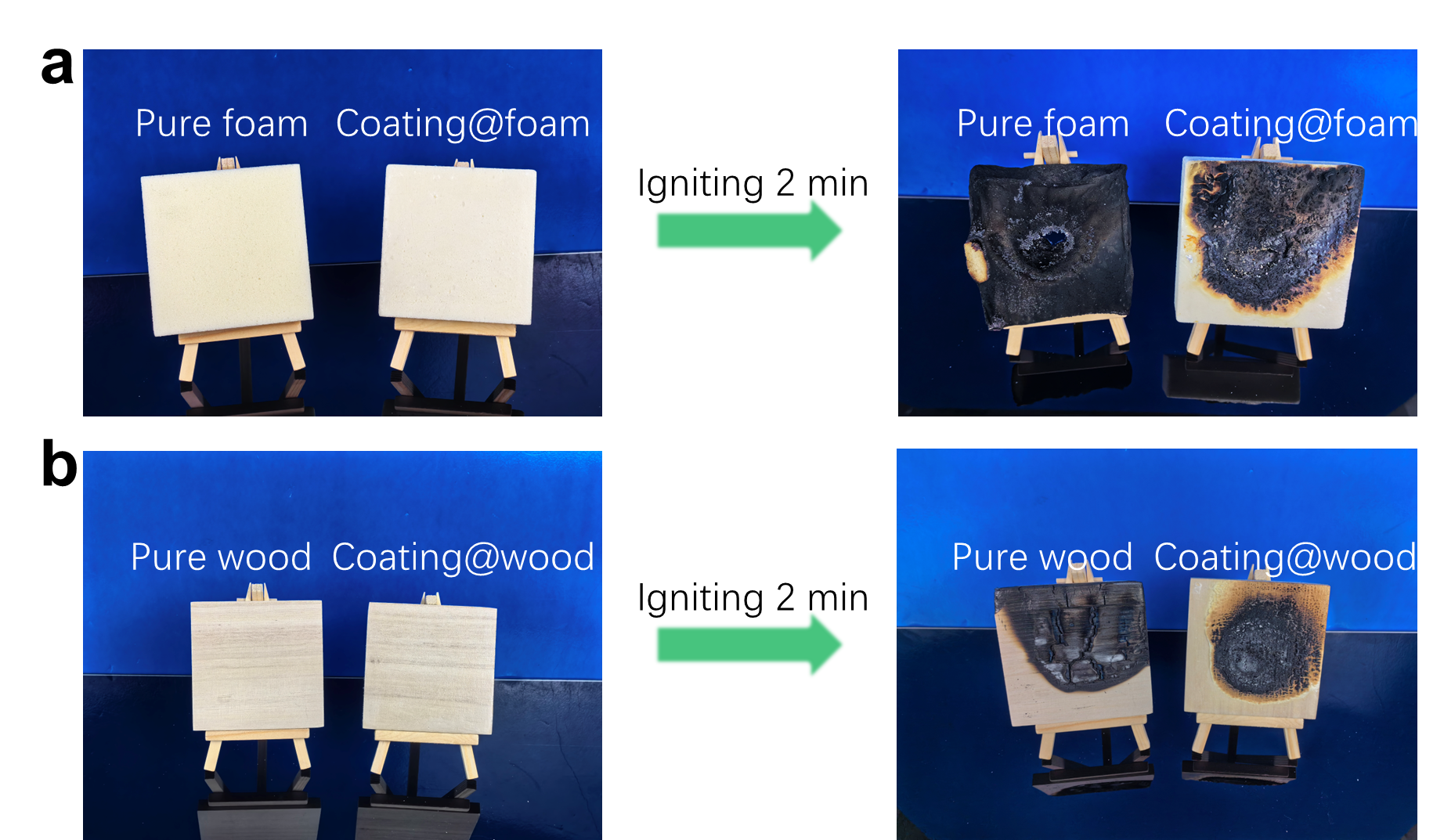
**

**Fig. S27** Digital images of pure wood and PBML@wood after burning under the butane flame for 120 s

**Table S1** Designation of the poly(SSS-co-HEA) with various SSS/HEMA ratios

| Polymer | SSS (mol) | HEMA (mol) | (NH_4_)_2_S_2_O_8_ (wt. %) |
| --- | --- | --- | --- |
| PSH-1 | 40 | 60 | 2 |
| PSH-2 | 50 | 50 | 2 |
| PSH-3 | 60 | 40 | 2 |

**Table S2** Designation and components of the hybrid coatings for various samples

|  | Composition (wt%) | | |  |  |
| --- | --- | --- | --- | --- | --- |
| Note | PSH | M⋅2B | LDH | Si-coatings | Transparency |
| PSH | 100 |  |  |  | Yes |
| PSH/LDH | 88.1 |  | 11.9 |  | Yes |
| PSH/BM | 89.6 | 10.4 |  |  | Yes |
| PSH/BM/LDH | 80 | 9.2 | 10.8 |  | Yes |
| PSH @wood | 100 |  |  |  | Yes |
| PSH/LDH@wood | 88.1 |  | 11.9 |  | Yes |
| PSH/BM @wood | 89.6 | 10.4 |  |  | Yes |
| PSH/BM/LDH@wood | 80 | 9.2 | 10.8 |  | Yes |
| Si-PSH/BM/LDH@wood | 80 | 9.2 | 10.8 | ~2 wt.% | Yes |
| PSH/BM/LDH@PU | 80 | 9.2 | 10.8 |  | Yes |

Note: Regardless of whether the amount of BM or LDH added exceeds the ratio written in the above table, the final samples will not remain transparent.

**Table S3** Signs of the main cross-peaks in 2Dcos synchronous and asynchronous spectra. Signs read in synchronous and asynchronous spectra (left: *Syn*; right: *Asyn*)

| 1077 | +, - | +, - | +, - | +, - | -, + | +, - | +, - |  |
| --- | --- | --- | --- | --- | --- | --- | --- | --- |
| 1121 | +, - | +, - | +, - | +, - | -, + | +, - |  |  |
| 1173 | +, - | +, - | +, - | +, - | -, + |  |  |  |
| 1280 | -, + | -, + | -, + | -, + |  |  |  |  |
| 1600 | +, - | +, + | +, + |  |  |  |  |  |
| 1636 | +, - | +, - |  |  |  |  |  |  |
| 1652 | +, - |  |  |  |  |  |  |  |
| 1716 |  |  |  |  |  |  |  |  |
|  | 1716 | 1652 | 1636 | 1600 | 1280 | 1173 | 1121 | 1077 |

Sign multiplication (*Syn*×*Asyn*)

| 1077 | - | - | - | - | - | - | - |  |
| --- | --- | --- | --- | --- | --- | --- | --- | --- |
| 1121 | - | - | - | - | - | - |  |  |
| 1173 | - | - | - | - | - |  |  |  |
| 1280 | - | - | - | - |  |  |  |  |
| 1600 | - | + | + |  |  |  |  |  |
| 1636 | - | - |  |  |  |  |  |  |
| 1652 | - |  |  |  |  |  |  |  |
| 1716 |  |  |  |  |  |  |  |  |
|  | 1716 | 1652 | 1636 | 1600 | 1280 | 1173 | 1121 | 1077 |

According to Noda’s rule (see Note S2), the order of different wavenumbers is: 1077 → 1121 → 1173 → 1280 → 1652 → 1636 → 1600 → 1716, i.e. *v*(C-OH) → *v*(B-O) → *v*(S=O) → *v*(B-OH) → *v*(N-H) → *v*(C=N) → *v*(C=O).

**Table S4** Percentage of each component in char after burning various times

|  |  | 10 s (%) | 30 s (%) | 300 s (%) |
| --- | --- | --- | --- | --- |
| B 1s | **B(OH)_3_** | 17.2% | 6.1% |  |
|  | **B_2_O_3_** | 38.5% | 43.7% | 17.8% |
|  | **B-O-C** | 44.3% |  |  |
|  | **B-C** |  | 26.4% | 27.4% |
|  | **BN** |  |  | 6.3% |
|  | **Na_2_B_4_O_7_** |  |  | 48.5% |
| C 1s | **C-C** | 60.9% | 30.8% | 32.6% |
|  | **C=C** | 1.2% | 12.8% | 26.3% |
|  | **C-SO_x_** | 37.9% | 45.8% | 33.0% |
|  | **Na_2_-CO_3_** |  | 10.6% | 8.2% |

**Table S5** TGA data of different film samples under air atmosphere

| Samples | T_5%_ ^a^ | T_max1_ ^b^ | T_max2_ ^c^ | CY800 |
| --- | --- | --- | --- | --- |
| PSH | 229 | 362 | 518 | 19 |
| PL | 175 | 445 | 578 | 18 |
| PBM | 222 | 399 | 582 | 18 |
| PBML | 215 | 447 | 585 | 24 |

^a^: Temperature at 5% weight loss; ^b^: T_max1_: Temperature at maximum thermal decomposition; ^c^: CY^800^: Residual rate at 800 °C

**Table S6** Cone calorimeter testing data for wood and various coated woods

| Samples | Wood | PSH | PL | PBM | PBML |
| --- | --- | --- | --- | --- | --- |
| t_ign_ (s) ^a^ | 14±1 | 378±4 | 444±5 | 392±3 | unignited |
| pHRR (kW/m^2^) ^a^ | 381±23 | 124±8 | 116±11 | 91±5 | 23±2 |
| t_pHRR_ (s) | 8±1 | 416±9 | 443±10 | 431±5 | 729±6 |
| THR (MJ/m^2^) ^a^ | 56±4 | 31±3 | 24±2 | 26±4 | 12±2 |
| pSPR (m^2^/m^2^) ^a^ | 0.021±0.002 | 0.044±0.005 | 0.027±0.003 | 0.047±0.002 | 0.019±0.003 |
| TSP (m^2^) ^a^ | 1.7±0.3 | 3.1±0.2 | 2.9±0.3 | 2.0±0.2 | 2.7±0.1 |
| Residue mass (%) ^a^ | 32±2 | 18±1 | 16±2 | 15±1 | 10±1 |
| COY (kg/kg) | 108±4 | 136±3 | 106±6 | 101±5 | 174±6 |
| CO_2_Y (kg/kg) | 1808±30 | 972±26 | 1332±31 | 1041±36 | 541±18 |
| FPI (m^2^s/kW) ^b^ | 0.037±0.003 | 3.039±0.03 | 3.838±0.4 | 4.327±0.3 | >38.662 |
| FGI (kW/m^2^/s) ^c^ | 47.70±3 | 0.30±0.02 | 0.26±0.02 | 0.21±0.01 | 0.03±0.003 |

^a^ t_ign_: time to ignition; pHRR: peak heat release rate; THR: total heat release; pSPR: peak smoke release rate; TSR: total smoke release; TSP: total smoke production; COY/ CO_2_Y: CO yield and CO_2_ yield.

^b^: FPI: fire performance index; FGI: fire growth index.

**Table S7** Toxicity of gases an emitted from smoke of various wood materials (ND is not detected)

| Samples | Wood | PSH | PL | PBM | PBML | Reference concentration (mg/m^3^) |
| --- | --- | --- | --- | --- | --- | --- |
| CO_2_ (ppm) | 1283±328 | 909±143 | 1074±195 | 1103±146 | 474±132 | 72,000 |
| CO (ppm) | 38.2±2.4 | 69.5±5.3 | 150.4±24.3 | 145.3±17.0 | 107.5±17.9 | 1380 |
| SO_2_ (ppm) | 13.4±1.3 | 9.9±1.41173 | 19.0±2.18364 | 18.4±1.30394 | 9.4±2.65405 | 262 |
| NO (ppm) | 1.3±0.35 | 1.0±0.14 | 0.9±0.10 | 1.1±0.22 | 0.3±0.08 | 38 |
| NO_2_ (ppm) | ND | ND | ND | ND | ND |  |
| HF (ppm) | ND | ND | ND | ND | ND | 25 |
| HCl (ppm) | 1.05±0.24 | 0.08±0.01 | 0.05±0.01 | 0.04±0.01 | 0.01±0.01 | 75 |
| HBr (ppm) | 0.26±0.03 | 0.27±0.01 | 0.21±0.03 | 0.25±0.02 | 0.25±0.02 | 99 |
| HCN (ppm) | 0.96±0.16 | 0.34±0.08 | 0.19±0.03 | 0.38±0.04 | 0.27±0.02 | 55 |
| CIT_G_ | 0.021±0.002 | 0.017±0.001 | 0.028±0.002 | 0.028±0.002 | 0.016±0.001 |  |

The conventional toxicity index (CIT_G_) consists principally of the ratios of measured concentrations of toxic smoke gas components to their reference concentrations, according to the formula:

$${CIT}_{G}=\frac{0.51m^{3}\times0.1m^{2}}{150m^{3}\times0.004225m^{2}}\times\sum_{i=1}^{i=8} \frac{c_{i}}{C_{i}}$$

where the model is the combustion of 0.1 m^2^ of product; the gaseous effluents are dispersed in 150 m^3^; the volume of the test chamber is 0.51 m^3^; the exposed surface of the test specimen is 0.004225 m^2^. c*_i_* and C*_i_* is the concentration measured in mg/m^3^ of the *i*^th^ gas in the smoke chamber according to EN ISO 5659-2 and the IDLH reference concentration in mg/m^3^ of the *i*^th^ gas. CIT_G_ values are dimensionless. The threshold value is 0.75 based on the standard (EN 45545-2), which means that values inferior to those could qualify the product for rail vehicles.

**Table S8** Combustible gases emitted from smoke of various wood materials

| Samples | Wood | PSH | PL | PBM | PBML |
| --- | --- | --- | --- | --- | --- |
| CH_2_O (ppm) | 1.0±0.18 | 4.7±0.39 | 8.1±1.31 | 7.1±1.43 | 4.7±1.63 |
| CH_4_ (ppm) | 2.9±0.45 | 16.4±0.36 | 27.0±3.40 | 27.5±3.47 | 23.4±3.67 |
| C_2_H_4_ (ppm) | 0.06±0.01 | 2.081±0.02 | 3.10±0.48 | 3.98±0.56 | 2.89±1.13 |
| C_3_H_6_ (ppm) | 2.29±0.60 | 4.14±0.56 | 6.01±1.23 | 8.60±1.01 | 5.35±2.65 |

**Table S9** A comparison of comprehensive properties with different MXene-based materials

| Film type ^a^ | Substrate ^b^ | LOI (%) | TTI/heat flux ^c^  (s/ kW⋅m^-2^) | pHRR (kW/m^2^) | THR (MJ/m^2^) | FGI (kW/m^2^⋅s) | CIT_G_ | FRI ^d^ | Refs. |
| --- | --- | --- | --- | --- | --- | --- | --- | --- | --- |
| DPDHPP@PU | PU | NG | 18/35 | 218.6  (-44.2%) | 19.5  (-13.7%) | NG | NG | NG | [S1] |
| PVH@PVH/BN/GP | PU | 35.8 | 21/25 | 226  (-34%) | 38 (33%) | 7.6 | NG | 9.51 | [S2] |
| PBM | PU | NG | 5/35 | 306.7 (+23.1%) | 34.6 (+2.9%) | NG | NG | 0.67 | [S3] |
| PDA/GO | PU | NG | NG/35 | 273.2  (-64.9%) | 21.4  (-12.3%) | NG | NG | NG | [S4] |
| F-MC | SiRF | 27.6 | 27.6/25 | 87.4  (-7.6%) | 54.1  (-17.9%) | NG | NG | NG | [S5] |
| APP/SWCNHs | Cotton | NG | Unignited/35 | 22.1  (-92.2%) | 3.35  (-58.4%) | 0.47 | NG | ∞ | [S6] |
| UCPR-LDHs | Wood | NG | 46/50 | 91.5  (-24.3%) | 33.6  (-26.2%) | NG | NG | 1.96 | [S7] |
| MCPR-LDHs | Wood | NG | 55/50 | 98.8  (-11.9%) | 39.1  (-20.3%) | NG | NG | 1.87 | [S7] |
| silica/polyurea | Wood | 48 | 65.2/NG | 121  (-45.9%) | 121  (-23.8%) | NG | NG | 11.01 | [S8] |
| PEI-GMWCNT/MMT | Wood | NG | 237/35 | 201  (-43.1%) | 68  (-44.7%) | 0.46 | NG | 15.40 | [S9] |
| PM-Fe^3+^/OA | Wood | 38.6 | 15/NG | 55.7  (-56.6%) | 2.1  (-52.2%) | 2.32 | NG | 10.5 | [S10] |
| MAW4 | Wood | 54.5 | 89/50 | 254  (-22.3%) | 54.9  (-29.5%) | NG | NG | 13.5 | [S11] |
| MPW-4 | Wood | 42.4 | 119/50 | 146.9  (-46.3%) | 32.2  (-30.8%) | NG | NG | 22.8 | [S12] |
| PSH/BM/LDH | **Wood** | **37.3** | **Unignited/50** | **23**  **(-94.0%)** | **12**  **(-78.6%)** | **0.03**  **(-99.4%)** | **0.016** | **>4417.4** | ***This work*** |

^a^ PVH/BN/GP: poly(2-hydroxyethyl acrylate -co-sodium vinylsulfonate)/boron nitride/low-melting glass powders; PBM: polyurethane acrylate resin/ melamine-based acrylate resin/(N, N-bis (2-hydroxyethyl acrylate) aminomethyl phosphonic acid diethyl ester (BHAAPE))/MXene; PDA/GO: polydopamine-graphene oxide; F-MC: 1, 1, 2, 2-tetrahydroperfluorodecyltrimethoxysilane /cellulose nanofibers/MXene; SiRF: polydimethylsiloxane foam; APP/SWCNHs: ammonium polyphosphate/carbonaceous nanomaterials-single-walled carbon nanohorns; MCPR/UCPR-LDHs: melamine-formaldehyde/urea-formaldehyde resin-layered double hydroxides; PEI-GMWCNT/MMT: polyethyleneimine/graphitized MWCNT/Na-montmorillonite sheets; PM-Fe^3+^/OA: melamine/phytic acid/anhydrous ferric chloride/octadecylamine; MAW4: melamine urea-formaldehyde resin/adenosine-based phosphonate; MPW: melamine-urea-formaldehyde/ammonium hydrogen phytate.

^b^ TTI: time to ignition. **^c^** FRI: flame Retardancy Index.

**3 Movies S1-S21**

Movie S1 Water bucket lifting process with PBML coating

Movie S2 Combustion processes of PSH film under butane torch flame attack

Movie S3 Combustion processes of PL film under butane torch flame attack

Movie S4 Combustion processes of PBM film under butane torch flame attack

Movie S5 Combustion processes of PBML film under butane torch flame attack

Movie S6 UL-94 testing of wood

Movie S7 UL-94 testing of PSH@wood

Movie S8 UL-94 testing of PL@wood

Movie S9 UL-94 testing of PBM@wood

Movie S10 UL-94 testing of PBML@wood

Movie S11 cone calorimeter testing of PBML@wood

Movie S12 Burning process for uncoated PU

Movie S13 Thermal video of burning process for uncoated PU

Movie S14 Burning process for coated PU

Movie S15 Thermal video of burning process for coated PU

Movie S16 Burning process for uncoated wood

Movie S17 Thermal video of burning process for uncoated wood

Movie S18 Burning process for coated wood

Movie S19 Thermal video of burning process for coated wood

Movie S20 Water-tolerant test of Si-PBML@wood

Movie S21 UL-94 testing of Si-PBML@wood

**Supplementary References**

1. D. Jiao, H. Sima, X. Shi, C. Zhang, B. Liu, Mussel-inspired flame retardant coating on polyurethane foam. Chem. Eng. J. **474**, 145588 (2023). <https://doi.org/10.1016/j.cej.2023.145588>
2. Z.W. Ma, J.Z. Zhang, C. Maluk, Y.M. Yu, S.M. Seraji et al., A lava-inspired micro/nano-structured ceramifiable org-anic-inorganic hybrid fire-extinguishing coating. Matter **5**(3), 911-932 (2022). <https://doi.org/10.1016/j.matt.2021.12.009>
3. Y.B. Huang, S.H. Jiang, R.C. Liang, P. Sun, Y. Hai et al., Thermal-triggered insulating fireproof layers: A novel fire-extinguishing MXene composites coating. Chem. Eng. J. **391**, 123621 (2020). <https://doi.org/10.1016/j.cej.2019.123621>
4. H. Kim, D.W. Kim, V. Vasagar, H. Ha, S. Nazarenko et al., Polydopamine-graphene oxide flame retardant nanocoatings applied via an aqueous liquid crystalline scaffold. Adv. Funct. Mater. **28**(39), 1803172 (2018). <https://doi.org/10.1002/adfm.201803172>
5. H.-Y. Chen, Z.-Y. Chen, M. Mao, Y.-Y. Wu, F. Yang et al., Self-adhesive polydimethylsiloxane foam materials decorated with MXene/cellulose nanofiber interconnected network for versatile functionalities. Adv. Funct. Mater. **33**(48), 2304927 (2023). <https://doi.org/10.1002/adfm.202304927>
6. J. Xu, Y. Niu, Z. Xie, F. Liang, F. Guo et al., Synergistic flame retardant effect of carbon nanohorns and ammonium polyphosphate as a novel flame retardant system for cotton fabrics. Chem. Eng. J. **451**, 138566 (2023). <https://doi.org/10.1016/j.cej.2022.138566>
7. C. Deng, Y. Liu, H. Jian, Y. Liang, M. Wen et al., Study on the preparation of flame retardant plywood by intercalation of phosphorus and nitrogen flame retardants modified with Mg/Al-LDH. Constr. Build. Mater. **374**, 130939 (2023). <https://doi.org/10.1016/j.conbuildmat.2023.130939>
8. T. Zhang, J. Xi, S. Qiu, B. Zhang, Z. Luo et al., Facilely produced highly adhered, low thermal conductivity and non-combustible coatings for fire safety. J. Colloid Interface Sci. **604**, 378-389 (2021). <https://doi.org/10.1016/j.jcis.2021.06.135>
9. X. Zhou, Q. Fu, Z. Zhang, Y. Fang, Y. Wang et al., Efficient flame-retardant hybrid coatings on wood plastic compo-sites by layer-by-layer assembly. J. Clean. Prod. **321**, 128949 (2021). <https://doi.org/10.1016/j.jclepro.2021.128949>
10. L. Ma, T. Zhang, Y. Zhao, T. Yuan, X. Wang et al., Preparation of multifunctional flame-retardant and superhydrophobic composite wood by iron ions doped phytic acid-based nanosheets. Constr. Build. Mater. **422**, 135854 (2024). <https://doi.org/10.1016/j.conbuildmat.2024.135854>
11. Q. Liu, H. Luo, Z. Gao, Y. Huang, J. Liang et al., Preparation of waterborne intumescent flame-retardant coatings us-ing adenosine-based phosphonates for wood surfaces. Prog. Org. Coat. **187**, 108061 (2024). <https://doi.org/10.1016/j.porgcoat.2023.108061>
12. F. Song, T. Liu, Q. Fan, D. Li, R. Ou et al., Sustainable, high-performance, flame-retardant waterborne wood coatings via phytic acid based green curing agent for melamine-urea-formaldehyde resin. Prog. Org. Coat. **162**, 106597 (2022). <https://doi.org/10.1016/j.porgcoat.2021.106597>
